# Adaptive Sparsening and Smoothing of the Treatment Model for Longitudinal Causal Inference Using Outcome‐Adaptive LASSO and Marginal Fused LASSO

**DOI:** 10.1002/sim.70316

**Published:** 2026-01-22

**Authors:** Mireille E. Schnitzer, Denis Talbot, Yan Liu, David Berger, Guanbo Wang, Jennifer O'Loughlin, Marie‐Pierre Sylvestre, Ashkan Ertefaie

**Affiliations:** ^1^ Faculté de pharmacie Université de Montréal Quebec Canada; ^2^ Département de médecine sociale et préventive Université de Montréal Quebec Canada; ^3^ Département de médecine sociale et préventive Université Laval Quebec Canada; ^4^ Centre de recherche du CHU de Québec Université Laval Quebec Canada; ^5^ Department of Computer Science Université de Montréal Quebec Canada; ^6^ The Dartmouth Institute for Health Policy and Clinical Practice Geisel School of Medicine at Dartmouth Hanover New Hampshire USA; ^7^ Department of Biomedical Data Science Geisel School of Medicine at Dartmouth Hanover New Hampshire USA; ^8^ Centre de recherche du centre hospitalier Université de Montréal Quebec Canada; ^9^ Department of Biostatistics, Epidemiology & Informatics, Division of Biostatistics University of Pennsylvania Philadelphia Pennsylvania USA

**Keywords:** causal inference, inverse probability weighting, LASSO, longitudinal data, marginal structural model, variable selection

## Abstract

Causal variable selection in time‐varying treatment settings is challenging due to evolving confounding effects. Existing methods mainly focus on time‐fixed exposures and are not directly applicable to time‐varying scenarios. We propose a novel two‐step procedure for variable selection when modeling the treatment probability at each time point. We first introduce a novel approach to longitudinal confounder selection using a Longitudinal Outcome Adaptive LASSO (LOAL) that will data‐adaptively select covariates with theoretical justification of variance reduction of the estimator of the causal effect. We then propose an adaptive fused LASSO that can collapse treatment model parameters over time points with the goal of simplifying the models in order to improve the efficiency of the estimator while minimizing model misspecification bias compared with naive pooled logistic regression models. Our simulation studies highlight the need for and usefulness of the proposed approach in practice. We implemented our method on data from the Nicotine Dependence in Teens study to estimate the effect of the timing of alcohol initiation during adolescence on depressive symptoms in early adulthood.

## 1 Introduction

Near practical positivity violations are a common problem when conducting causal inference with time‐varying binary treatments, especially when treatment can change over many time points. For marginal structural models (MSMs), which are models for the counterfactual outcome under a longitudinal treatment intervention, the typical sequential positivity assumption requires that, at each time point, the probability of accessing either level of treatment (propensity score) is nonzero for any possible covariate values and prior treatments. The related practical condition is that one must have observed outcome information under all relevant treatment patterns and baseline and time‐varying covariate values. In typical finite sample applications, this condition is difficult to satisfy [1, 2]. Under such data sparsity, outcome regression‐based methods can smooth over time points where treatments are not observed for certain covariate strata. However, unless all outcome models are correctly specified—which is difficult to achieve when extrapolation is necessary—this approach leads to biased estimation. Alternatively, many methods involve weighting by the inverse of the probability of treatment [3, 4, 5]. This involves directly modeling the probability of treatment for each time‐point. When smoothing is desirable, it is possible to pool these treatment models over time [1, 6, 7] or covariate information [6], but this can also lead to bias if the resulting models are not correctly specified for the probability of treatment at each time‐point. Doubly robust causal inference approaches such as Targeted Maximum Likelihood Estimation [5, 8] (TMLE), Augmented‐Inverse Probability of Treatment Weighting [3], and Double Machine Learning [9], which can leverage machine learning methods for the estimation of nuisance quantities, perform well in the absence of data sparsity. However, while they avoid parametric assumptions, machine learning applied directly to estimating the probability of treatment may exacerbate issues related to sparsity [10, 11]. It is, therefore, of interest to develop data‐adaptive approaches to model selection that can trade off bias and variance under sparse conditions.

A related challenge involves defining and selecting covariates to satisfy the sequential conditional exchangeability (“no unmeasured confounders”) assumption [6]. Beyond adjustment for confounders, excluding covariates that only affect the treatment (i.e., instruments) and adjusting for pure causes of the outcome can reduce estimation variance with inverse probability of treatment weighting [12, 13, 14]. Previous work related to fitting MSMs with time‐varying treatments demonstrated quantitatively [15] and theoretically [16, 17] that estimation variance can be inflated when adjusting for variables that only cause treatment. In particular, Rotnitzky and Smucler (2020) [16] established that, under a nonparametric model with a given directed acyclic graph (DAG), and a time‐dependent adjustment set, the removal of certain types of non‐confounding covariates will reduce the asymptotic variance of nonparametric efficient estimators.

Many approaches for covariate selection or reduction that target reduced variance of the causal estimator have been developed in a single‐time‐point treatment setting [18, 19, 20, 21], including Bayesian approaches [22, 23, 24]. In particular, Shortreed and Ertefaie's outcome‐adaptive LASSO [25] uses the inverse magnitude of the outcome model regression coefficients to penalize the corresponding covariates in an adaptive LASSO for the treatment model. This aims to exclude any variable from the treatment model that does not have a conditional association with the outcome. One version of Collaborative Targeted Maximum Likelihood Estimation (C‐TMLE) [26] greedily selects covariates into the treatment model when the inclusion improves the fit of the propensity score‐updated outcome model, and uses cross‐validation to select an optimal number of selection steps. In the longitudinal setting, there are few options for dealing with data sparsity. Schnitzer et al. (2020) [27] extended C‐TMLE to the longitudinal treatment setting but noted its computational complexity. Other approaches [28, 29] have been developed that modify the target causal parameter, leading to a different scientific interpretation, but allowing for greater robustness to sparsity.

While not yet applied to any causal setting, the fused LASSO [30, 31, 32] was proposed to smooth over spacial or temporal structures by penalizing both coefficient magnitudes and the distance between coefficients of neighboring or grouped covariates in a linear regression model. Viallon et al. [32, 33] proposed an extension for generalized linear models with adaptive weights [34] resulting in oracle properties. The optimization problem is solved with a coordinate‐wise optimization algorithm [30].

In this study, we explore a two‐phase approach to treatment model dimension reduction in the longitudinal context with a causal objective of estimating the parameters of an MSM. We first define a “saturated” model for the probability of treatment at all time‐points that adjusts for the complete history of covariates and completely stratifies the model by time. Then, we propose to remove covariates from this model using a longitudinal extension of the outcome‐adaptive LASSO applied to the saturated model. Our selection criteria use parametric working outcome models to operationalize the variance reduction results [16, 17]. The second step, carried out after the initial covariate reduction, involves model smoothing over time such that covariate‐treatment associations can have shared parameters over time. This step utilizes an implementation of the adaptive fused LASSO for a logistic regression that penalizes the distance between the coefficients of a given covariate and the treatment at different time points. We then perform extensive simulation studies to evaluate the performance and robustness of our approach compared to non‐adaptive and oracle estimators and longitudinal C‐TMLE. Finally, we apply our approach in a complex longitudinal setting to estimate the effect of drinking initiation in adolescents on scores of depression symptoms in early adulthood.

## 2 Data, Target Parameter, and Estimation

In this section, we present the target of estimation and give preliminaries that will allow us to describe our proposed model selection procedure.

### 2.1 Data and Target Parameter

Suppose that we have a data structure $O=(L_{0},A_{0},L_{1},A_{1},\ldots ,A_{T},Y)$ where the outcome Y is continuous, the treatments $A_{t};t=0,\ldots ,T$ are binary, and the covariates $L_{t};t=0,\ldots ,T$ are multivariate. We use ${\bar{L}}_{t}=(L_{0},\ldots ,L_{t})$ to indicate the history of covariates up to and including time t. The covariates potentially include both binary and continuous components. We take n independent identically distributed samples, with realizations denoted by lowercase letters, for example, $o_{i}$ and $a_{0,i}$ are realizations of O and $A_{0}$, respectively, for i=1,…,n.

We consider an intervention that sets each treatment node $A_{t}$ to some fixed value, either zero or one, that is, setting $\bar{A}=(A_{0},\ldots ,A_{T})$ to the treatment pattern $\bar{a}=(a_{0},\ldots ,a_{T})$ where each $a_{t}$ is either zero or one. Define the counterfactual variable $Y^{\bar{a}}$ to be the potential outcome under treatment pattern $\bar{a}$. Our interests lie in modeling the marginal expectation of the counterfactual outcome potentially conditional on some subset of the baseline covariates $L_{0}$, that is, modeling $𝔼(Y^{\bar{a}}|L_{0})$. As an example, define the MSM of interest as

(1) $$\begin{array}{l} 𝔼(Y^{\bar{a}}|L_{0}^{1})=\mu_{0}+\mu_{1}L_{0}^{1}+\mu_{2}cum(\bar{a}), \end{array}$$

where $L_{0}^{1}\subseteq L_{0}$ is a single (one‐dimensional) baseline covariate, and $cum(\bar{a})$ represents the cumulative function that counts the number of ones (i.e., number of treated time‐points) in the treatment pattern $\bar{a}$. Thus, $\mu_{2}$ represents the change in the expected outcome from one additional treated time point under the linear model, and $\left\{\mu_{0},\mu_{1},\mu_{2}\right\}$ is our target parameter. We can more appropriately define the MSM as a working model, and the true parameter values as projections of the true counterfactual regression curve $𝔼(Y^{\bar{a}}|L_{0}^{1})$ onto the working model under a given loss function [35]. See Petersen et al. [36] for complete details in the time‐varying treatment context.

### 2.2 Identifiability

The MSM parameters are identifiable under typical causal assumptions, including consistency, sequential positivity, and sequential conditional exchangeability [37]. Sequential conditional exchangeability is given as

$$\begin{array}{l} Y^{\bar{a}}\;⊥\;A_{t}|({\bar{A}}_{t-1}={\bar{a}}_{t-1},{\bar{L}}_{t}),\;\;t=0,\ldots ,T, \end{array}$$

where variables with negative subscripts should be discarded here and subsequently. Positivity means that $P(A_{t}|{\bar{L}}_{t},{\bar{A}}_{t-1})>0$ for all supported values of $\left({\bar{L}}_{t},{\bar{A}}_{t-1}\right)$. Consistency means that we can equate the potential outcome under the observed treatment to the observed outcome, that is, $Y^{\bar{a}}=Y$ if $\bar{A}=\bar{a}$.

In order to review the identifiability of the MSM parameters, we define nested expectations of the outcome conditional on the treatment pattern of interest [3]. We initialize $q_{T+1}({\bar{a}}_{T+1},{\bar{L}}_{T+1})=Y$. We recursively define

$$\begin{array}{l} q_{t}({\bar{a}}_{t},{\bar{L}}_{t})=𝔼\{q_{t+1}({\bar{a}}_{t+1},{\bar{L}}_{t+1})|{\bar{A}}_{t}={\bar{a}}_{t},{\bar{L}}_{t}\},\;\;t=T,\ldots ,0. \end{array}$$

We will use the notation $q_{t}^{\bar{a}}=q_{t}(\bar{a},{\bar{L}}_{t})$ for brevity. Under the above causal assumptions, the g‐formula $𝔼(q_{0}^{\bar{a}}|L_{0}^{1})=𝔼(Y^{\bar{a}}|L_{0}^{1})$ identifies the true regression curve for $Y^{\bar{a}}$ with respect to $\bar{a}$ and $L_{0}^{1}$ [3]. Under the MSM in Equation (1), this also identifies the true values of the parameters $\mu =(\mu_{0},\mu_{1},\mu_{2})$.

If we do not want to make any causal assumptions, we can alternatively define our parameter of inference statistically through the function $q_{0}^{\bar{a}}$. The parameters μ can be directly defined as minimizing the least‐squares risk function of the model with $q_{0}^{\bar{a}}$ as the outcome with a regression specification according to the right‐hand side of Equation (1).

### 2.3 Estimators

There are many available estimators of μ. One such estimator is G‐computation, which uses regressions to sequentially estimate each $q_{t}^{\bar{a}}$, starting at time T [3, 38]. Then, define $q_{t}^{s}$ to be the vector composed of the stacked quantities $q_{t}^{\bar{a}}$ of each possible pattern $\bar{a}$. The final step involves regressing the estimate of the stacked vector $q_{0}^{s}$ according to the MSM in Equation (1).

Inverse probability of treatment weighting (IPTW) is an alternative approach that involves estimating the functions $g_{t}({\bar{a}}_{t},{\bar{L}}_{t})=P(A_{t}=a_{t}|{\bar{L}}_{t},{\bar{A}}_{t-1}={\bar{a}}_{t-1})$ for each time‐point t=0,…,T. One implementation then involves regressing the observed Y on the covariates in Model (1) using weights equal to estimates of $w_{t}({\bar{a}}_{t},{\bar{L}}_{t})=\prod_{k=0}^{t}\frac{g_{k}(a_{k},L_{0}^{1})}{g_{k}({\bar{a}}_{k},{\bar{L}}_{k})}$ for t=T, where $g_{t}(a_{t},L_{0}^{1})$ is defined as the stabilizing probability $P(A_{t}=a_{t}|L_{0}^{1})$. Longitudinal targeted maximum likelihood estimation (LTMLE) [5, 36, 38] is an approach that uses estimates of the functions $q_{t}^{\bar{a}}$ in addition to the weights $w_{t}({\bar{a}}_{t},{\bar{L}}_{t})$ in order to estimate the parameters of the MSM.

A primary question across all methods that use inverse probability of treatment weights (such as IPTW and LTMLE) is how to approach modeling the treatment process to estimate the functions $g_{t}({\bar{a}}_{t},{\bar{L}}_{t})$. The two primary approaches are to model the treatment separately at each time‐point or to pool the model over the T+1 time‐points. The latter is interesting because it allows for model simplification under sparsity while still allowing for greater model complexity with sufficient data support. But without a priori restrictions on the conditioning of the pooled treatment model, it is clear that the number of covariates can become large as the number of time‐points increases. And indeed, incorrect pooling and modeling decisions can lead to bias in the estimation of the MSM parameters.

## 3 Variable Selection

In this section, we describe covariate reduction of the treatment model using an implementation of the outcome‐adaptive LASSO.

### 3.1 Selection Goal

We consider parametric logistic regressions for the treatment models. For simplified illustration, we consider two time‐points (T=1), with the associated data structure $O=(L_{0},A_{0},L_{1},A_{1},Y)$. We consider a model for the probability of treatment, stratified on time, written as

(2) $$\begin{array}{ll} \text{logit}\left\{P(A_{0}=1|L_{0})\right\} & =\alpha_{0,-1}+\alpha_{0,0}L_{0}, \end{array}$$

(3) $$\begin{array}{ll} \text{logit}\left\{P(A_{1}=1|{\bar{L}}_{1},A_{0})\right\} & =\alpha_{1,-1}+\alpha_{1,0}L_{0}+\alpha_{1,1}L_{1}+\alpha_{1,2}A_{0}, \end{array}$$

where the coefficients may be vectors when the corresponding $L_{t}$ is multivariate. We can represent the same restriction on the mean as the pooled model

(4) $$\begin{array}{ll} m_{t}({\bar{L}}_{t},{\bar{A}}_{t-1};\alpha ) & =P(A_{t}=1|{\bar{A}}_{t-1},{\bar{L}}_{t})={\text{logit}}^{-1}\left\{𝕀(t=0)\left(\alpha_{0,-1}\right.\right.\\ & \;\left.+\;\alpha_{0,0}L_{0}\right)+𝕀(t=1)\left(\alpha_{1,-1}+\alpha_{1,0}L_{0}+\alpha_{1,1}L_{1}\right.\\ & \;\left.\left.+\;\alpha_{1,2}A_{0}\right)\right\}, \end{array}$$

where t∈{0,1} and 𝕀(·) is the indicator function.

Define $\alpha =(\alpha_{0,-1},\ldots ,\alpha_{1,2})$, which are the coefficients of the covariates in the pooled propensity score model in (4).

Now consider the working regression models

(5) $$\begin{array}{ll} & E(q_{0}^{\bar{a}}|L_{0})=\beta_{0,-1}+\beta_{0,0}L_{0}, \end{array}$$

(6) $$\begin{array}{ll} & E(q_{1}^{\bar{a}}|{\bar{L}}_{1})=\beta_{1,-1}+\beta_{1,0}L_{0}+\beta_{1,1}L_{1}+\beta_{1,2}a_{0}, \end{array}$$

with true parameter values minimizing the risk under a squared‐error loss function. Note that under the causal assumptions, these correspond to working structural models for $Y^{\bar{a}}$, that is, Model (5) for $E(Y^{\bar{a}}|L_{0})$ and Model (6) for $E(Y^{\bar{a}}|{\bar{L}}_{1})$. Denote $\beta =(\beta_{0,-1},\ldots ,\beta_{1,2})$ and let $\beta^{†}=(\beta_{0,-1}^{†},\ldots ,\beta_{1,2}^{†})$ be an indicator vector of the nonzero elements of β, fixing the intercept and treatment terms as nonzero, that is, $\beta_{0,-1}^{†}=\beta_{1,-1}^{†}=\beta_{1,2}^{†}=1$.

We characterize the specific objectives of our variable selection as:

*For each time‐point, select variables into the treatment model at time* t *that have corresponding nonzero coefficients* β *in the model for* $q_{t}^{\bar{a}}$ *. We estimate the coefficients of the propensity scores*

(7) $$\begin{array}{ll} & m_{t}({\bar{L}}_{t},{\bar{A}}_{t-1};\alpha^{†}),\;t=0,1,\; \end{array}$$

*where* $\alpha^{†}$ *, of the same length as* α *, has fixed elements equal to zero corresponding to the zero items in* $\beta^{†}$ *. The optimal value of* $\alpha^{†}$ *, denoted as* $\alpha_{0}^{†}$ *(with the same elements fixed at zero), minimizes the risk under the logistic quasi‐log‐likelihood loss function*.

This specific variable selection criterion can be motivated as removing covariates from the adjustment set ${\bar{L}}_{T}$ that are not associated with the potential outcome $Y^{\bar{a}}$ conditional on the remaining variables in ${\bar{L}}_{T}$, and the past treatment ${\bar{A}}_{T-1}$. In particular, these covariates are not relevant for sequential conditional exchangeability (i.e., are not confounders). However, the criterion retains variables that are conditionally associated with the potential outcome, regardless of whether they are confounders. This is an operationalization of the identification of a covariate subset that, when removed from the adjustment set, leads to a reduction in estimation variance in the nonparametric model [16, 17]. Objective 1 also corresponds with the recommendations in [15] to only adjust for variables that affect the outcome through pathways that do not include treatment.

### 3.2 Longitudinal Outcome Adaptive Lasso (LOAL)

In order to write out the estimator, we expand the notation of the possibly multivariate covariates. First, we use τ=0,1 to index the propensity score model for $A_{\tau }$ (i.e., Models (2) and (3)). We use t=0,1 to index the covariates $L_{t}$ as before, where $L_{0}\in {\mathbb{R}}^{p_{0}}$ and $L_{1}\in {\mathbb{R}}^{p_{1}}$ such that $p_{t}$ is the number of covariates of $L_{t}$. The $k^{\text{th}}$ component of $L_{t}$ is denoted $L_{t,k};k=1,\ldots ,p_{t}$. Denote the set $𝒥=\{(0,1,{𝒥}_{0,0}),(1,1,{𝒥}_{1,0}),(1,2,{𝒥}_{1,1})\}$ as the 3‐dimensional indices of the coefficients α being shrunk, where the set ${𝒥}_{\tau ,t}$ indexes the specific covariates in $L_{t}$ being shrunk within propensity score model τ. Note that overlapping indices in ${𝒥}_{0,0}$ and ${𝒥}_{1,0}$ index coefficients for the same covariates in different models. For example, (0,0,1) and (1,0,1) refer to the coefficients of covariate $L_{0,1}$ in Models (2) and (3), respectively, or the equivalent in Model (4). Also note that the intercept coefficients (indices (0,−1) and (1,−1) in Model (4)), as well as the coefficients associated with treatment (index (1,2) in Model (4)) are not candidates for shrinking and so are excluded from 𝒥. The indices in 𝒥 are similarly used to refer to the corresponding coefficients β in Models (5) and (6).

Suppose that we have estimates $\hat{\beta }$ of β in Models (5) and (6) that are $\sqrt{n}$‐consistent where n is the sample size. For instance, we might estimate the functions $q_{0}^{ā}$ and $q_{1}^{ā}$ with correctly specified parametric models and then regress these quantities onto $L_{0}$ and ${\bar{L}}_{1}$, respectively. Given a regularization parameter $\lambda_{n}\geq 0$, an outcome‐adaptive LASSO estimator of $\alpha^{†}$ in the pooled Model (4) as defined in Objective 1 is given as

$$\begin{array}{ll} \hat{\alpha }(\lambda_{n}) & =\underset{\alpha }{\text{arg min}}\;\overset{1}{\underset{\tau =0}{\sum }}\overset{n}{\underset{i=1}{\sum }}\left[a_{\tau ,i}\log\{m_{\tau }({\bar{l}}_{\tau ,i},{\bar{a}}_{\tau -1,i};\alpha )\}\right.\\ & \;\left.+\;(1-a_{\tau ,i})\log\{1-m_{\tau }({\bar{l}}_{\tau ,i},{\bar{a}}_{\tau -1,i};\alpha )\}\right]+\lambda_{n}\underset{j\in 𝒥}{\sum }{\hat{\omega }}_{j}|\alpha_{j}|, \end{array}$$

where ${\hat{\omega }}_{j}=|{\hat{\beta }}_{j}{|}^{-\gamma }$ for all j∈𝒥, with tuning parameter γ>1. By the results in [25], this estimator is asymptotically normal and consistent for the selection of covariates in the Model (7) if we assume that $\lambda_{n}/\sqrt{n}\to 0$ and $\lambda_{n}n^{\gamma /2-1}\to \infty$. Note that γ>2 is needed for the second convergence requirement.

For implementation purposes, this regularized regression can be run using a transformation of the pooled data, setting $V_{0,-1}=1-𝕀(t=1)$, $V_{0,0}=L_{0}-𝕀(t=1)L_{0}$, $V_{1,-1}=𝕀(t=1)$, $V_{1,0}=𝕀(t=1)L_{0}$, $V_{1,1}=𝕀(t=1)L_{1}$, and $V_{1,2}=𝕀(t=1)A_{0}$ with corresponding coefficients $\alpha_{0,-1},\ldots ,\alpha_{1,2}$ in Model (4). Then, the adaptive LASSO is run with pooled outcome $A_{t}$ on covariates $V_{0,-1},\ldots ,V_{1,2}$, without an intercept term, using weights ${\hat{\omega }}_{j}=|{\hat{\beta }}_{j}{|}^{-\gamma };\forall j\in 𝒥$.

### 3.3 Estimation of $q_{t}^{\bar{a}}$ to Estimate β

The proposed variable selection for the propensity scores is based on the estimated β parameters in Models (5) and (6), which need to be estimated at $\sqrt{n}$ rates. However, this requires preliminary estimates of $q_{1}^{\bar{a}}$ and $q_{0}^{\bar{a}}$. To get these, we could first use a flexible regression method to estimate $q_{1}^{\bar{a}}$ by regressing Y on ${\bar{L}}_{1}$ and ${\bar{A}}_{1}$. We then generate predictions from this model for each pattern of interest ${\bar{a}}_{1}=(a_{0},a_{1})$. We then run a pooled regression of the stacked vector $q_{1}^{s}$ on the covariates ${\bar{L}}_{1}$ and $a_{0}$ where $a_{0}$ takes the value zero or one depending on the pattern ${\bar{a}}_{1}$, corresponding to the working structural Model (6). This results in estimates of the coefficients in that model, denoted by ${\hat{\beta }}_{1,-1},\ldots ,{\hat{\beta }}_{1,2}$.

**FIGURE 1:**
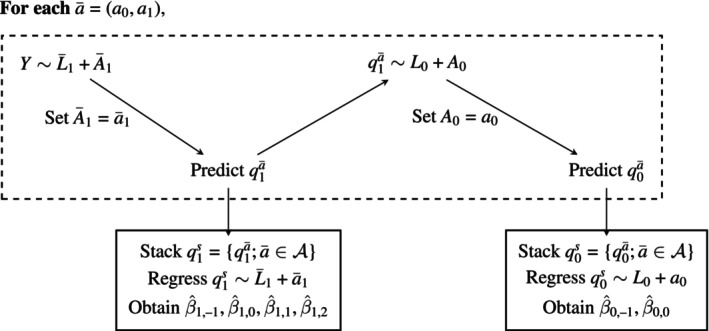
Diagram illustrating the steps required to estimate $q_{t}^{ā}$ and β, as described in Section 3.3.

For each pattern $\bar{a}\in 𝒜$ where 𝒜 is the set of all possible patterns, use a flexible regression method to regress $q_{1}^{\bar{a}}$ on $L_{0}$ and $A_{0}$. We then use this model to make predictions setting $A_{0}=a_{0}$ to obtain $q_{0}^{\bar{a}}=𝔼(q_{1}^{\bar{a}}|L_{0},a_{0})$. We then run a pooled regression of the stacked vector $q_{0}^{s}$ on the covariate $L_{0}$ according to the structural Model (5) to obtain estimates of $\beta_{0,-1}$ and $\beta_{0,0}$, which we will denote ${\hat{\beta }}_{0,-1}$ and ${\hat{\beta }}_{0,0}$, respectively. For illustration, each estimation step is presented in the Figure 1.

### 3.4 Selection of the Tuning Parameters

To select values for the two tuning parameters of the LOAL, we first fix the value of γ to a value slightly larger than 2 (we used 2.5 in the simulation and application) in order to ensure the required divergence of $\lambda_{n}n^{\gamma /2-1}$. We allow for a wide range of candidate $\lambda_{n}$ values such that the largest value shrinks all coefficients to zero. We propose to select $\lambda_{n}$ using a one‐dimensional extension of the weighted absolute mean difference proposed in Shortreed and Ertefaie [25], corresponding to a summary of a longitudinal balancing metric over covariates and times. See Web Appendix A for details. Note that in their simulation study, Shortreed and Ertefaie [25] selected γ such that $\lambda_{n}n^{\gamma /2-1}=n^{2}$ and set the range of values $\lambda_{n}=(n^{ℵ})$ with ℵ<0.5 to ensure both convergence requirements. In either approach, γ>2 and the balance criterion used to select $\lambda_{n}$ produces a tradeoff between covariate balance and model fit.

## 4 Selective Fusion

In this section, we describe a second approach to dimension reduction that adaptively pools related coefficients across the time‐point‐specific treatment models using the fused LASSO.

### 4.1 Fusion Goal

The Model (7) may not be sufficiently parsimonious in the following situation: suppose that, for some k, $\beta_{0,0,k}$ and $\beta_{1,0,k}$, the coefficients of covariate $L_{0,k}$ in the two structural models, are both large. This will mean that little penalty will be placed on the coefficients $\alpha_{0,0,k}$ and $\alpha_{1,0,k}$ in the LOAL procedure. In the situation where, for some $k\in {𝒥}_{0,0}\cap {𝒥}_{1,0}$, there is little difference in log‐odds between $L_{0,k}$ and $A_{1}$ relative to $L_{0,k}$ and $A_{0}$, conditional on other terms in the model, we want the LOAL to fuse the terms $\alpha_{0,0,k}$ and $\alpha_{1,0,k}$. That is, we want to set $\alpha_{0,0,k}=\alpha_{1,0,k}$ or equivalently, have a single time‐independent coefficient for $L_{0,k}$. This has the effect of smoothing over time and is the finite‐sample objective 2.

*In finite samples, fuse coefficients for common covariates across treatment models at different time points if it improves the pooled treatment model fit*.

By reducing the number of degrees of freedom and avoiding potential overfitting of the propensity score models, we expect that this objective will lead to more efficient estimation of μ in finite samples and avoid non‐data‐driven smoothing decisions under data sparsity.

### 4.2 Estimation

In order to achieve objective 2, we first obtain the estimates ${\hat{\alpha }}^{\text{refit}}(\lambda_{n})$ from the LOAL and a refitted logistic regression, and define $\alpha^{*}$ as the parameter vector of the same length as α that is set to zero at the indices of the zero‐elements of ${\hat{\alpha }}^{\text{refit}}(\lambda_{n})$. Then we use a generalized Adaptive Fused LASSO [32],

$$\begin{array}{ll} {\text{arg min}}_{\alpha^{*}}\overset{1}{\underset{\tau =0}{\sum }} & \overset{n}{\underset{i=1}{\sum }}\left[a_{\tau ,i}\log\{m_{\tau }({\bar{l}}_{1,i},a_{0,i};\alpha^{*})\}+(1-a_{\tau ,i})\right.\\ & \times \left.\log\{1-m_{\tau }({\bar{l}}_{1,i},a_{0,i};\alpha^{*})\}\right]\\ & +\lambda_{1,n}\underset{k\in {𝒥}_{0,0}^{*}\cap {𝒥}_{1,0}^{*},}{\sum }\frac{\left|\alpha_{1,0,k}^{*}-\alpha_{0,0,k}^{*}\right|}{{\left|{\hat{\alpha }}_{1,0,k}^{\text{refit}}(\lambda_{n})-{\hat{\alpha }}_{0,0,k}^{\text{refit}}(\lambda_{n})\right|}^{\gamma_{1}}}, \end{array}$$

with $\gamma_{1}>0$ and where ${𝒥}_{0,0}^{*}\subset {𝒥}_{0,0}$ and ${𝒥}_{1,0}^{*}\subset {𝒥}_{1,0}$ represent the indices of the selected covariates at τ=0 and 1, respectively. We propose to select the tuning parameters $\gamma_{1}$ and $\lambda_{1,n}$ by Bayesian information criterion (BIC) [32]. We omit a sparsity‐inducing penalty for two reasons: our variable selection was performed in the separate first step, and the variable selection and fusion objectives have different statistical goals (covariate balance vs. model selection, respectively).

It is important to note that, due to noncollapsibility and collinearity, the values of the coefficients in the pooled treatment models depend on the other covariates in the model. So, we first need to identify the covariate set before being able to statistically determine whether or not two coefficients should be fused. Thus, our application of the adaptive fused LASSO with a logistic regression model involves a purposeful misspecification of the pooled treatment model, where we have already potentially marginalized over covariates in the previous step. We define a graph over the remaining covariates indicating which we allow to fuse. The oracle results of Viallon et al. (2016) [32] apply relative to the model marginalized over the covariates removed in the first step, assuming that the treatment follows a Bernoulli distribution with a mean that is logit‐linear in the remaining covariates. We also need that ${\hat{\alpha }}^{\text{refit}}(\lambda_{n})$ converges to $\alpha^{†}$ at a $\sqrt{n}$‐rate, which is supported by the LOAL theory. Our application of the adaptive fused LASSO does not include a sparsity component in the objective function (i.e., does not include a LASSO penalty for the sizes of the coefficients). Through the same arguments, the oracle results then hold for the fusion of equal parameters $\alpha_{k}$ that are connected in the graph. A formal statement is given in Web Appendix B.

### 4.3 Fusion With T>1

This procedure can be expanded when the number of time‐points is greater than 2. The Fused LASSO requires the user to define a graph indicating which coefficients are allowed to fuse [32]. This graph should represent the maximum smoothing of the model through data pooling, corresponding perhaps to how propensity score models are typically pooled. For baseline covariates, a “clique graph” may be used where we allow the coefficients of common variables to fuse between times τ=0,…,T. Alternatively, we may use a “chain graph” where each chain connects the coefficient of the same baseline variable at successive time points. For common time‐updated covariates, one may fuse coefficients of variables with the same lag relative to the treatment time τ across times τ, for which we could use a chain graph for successive fusing or a clique graph for fusing between any two time‐points. We illustrate the usage of clique graphs with lagged time‐dependent variables in the application.

## 5 Simulations

In this simulation study, we estimated the coefficients in the MSM (1) where $L_{0}^{1}$ was always defined as the first confounder in the dataset. We applied LOAL as described in Section 3 and the two‐step fused LOAL (LOAL followed by the fusion step described in Section 4) to estimate the three target parameters in the MSM (1) using IPTW. For benchmarks, we also ran the sequential G‐computation [3] with all covariate main terms, IPTW with treatment models stratified by time‐point and including all covariate main terms (“full IPTW”), and IPTW with pooled treatment models excluding all unwanted covariates (“IPTW oracle select”) and further with correctly fused coefficients for common terms (“IPTW oracle select and fuse”). For fair comparisons, the specification of the models for estimating each $q_{t}$ was common across methods that used these quantities.

The LOAL was implemented using adaptive weights in glmnet [39]. We set γ=2.5 (which allows for the convergence of $\lambda_{n}n^{\gamma /2-1}$) and a very broad range for candidate λ values and then selected the optimal $\lambda_{n}$ value according to the balance criterion (Web Appendix A). We performed the fusion step using the archived FusedLasso package [40] which implements the coordinate‐wise optimization algorithm of Höfling, Binder, and Schumacher (2010) [30], implementing adaptive weights for fusion [32] but setting the adaptive weights for the main terms to zero so that no additional sparsity would be induced. We set $\gamma_{1}=2.5$ and searched over a very broad range for $\lambda_{1}$, selecting the optimal $\lambda_{1,n}$ using the BIC.

### 5.1 Evaluation in a Simple Scenario

**FIGURE 2:**
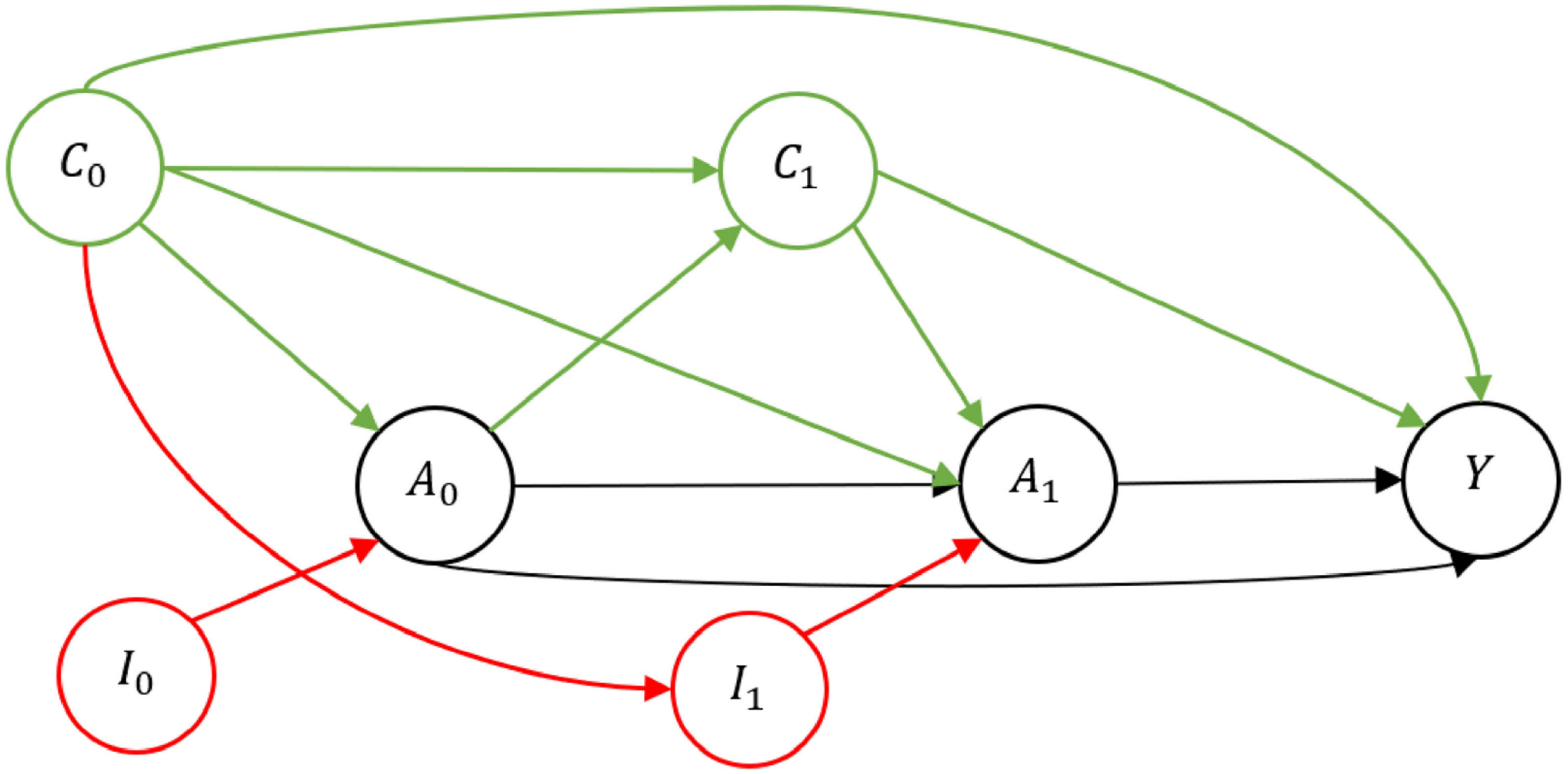
DAG representing the data generation in Scenario 1. The target variable selection retained all covariates labeled C.

In the simple scenario (Scenario 1), we generated i.i.d. data $O=(C_{0},I_{0},A_{0},C_{1},I_{1},A_{1},Y)$ according to the left DAG in Figure 2, where $C_{0}$, and $I_{0}$ were independently generated from a standard normal distribution and $A_{0}$ and $A_{1}$ were Bernoulli distributed. Variables $C_{1}$ and $I_{1}$ were Gaussian‐distributed with means $\left(A_{0}+C_{0}\right)$ and $\left(C_{0}\right)$, respectively. The instruments $I_{0}$ and $I_{1}$ only affected the treatment probabilities. Notably, $A_{0}$ was equal to one with probability ${\text{logit}}^{-1}(1.515C_{0}+I_{0})$ and $A_{1}$ with probability ${\text{logit}}^{-1}(-0.5+0.5C_{0}+0.25C_{1}+0.5A_{0}+I_{1})$. This made it so that in the marginal treatment models without instruments, the coefficients of $C_{0}$ in each model were both equal to 1.28. No other coefficients were equal. All four covariates were standardized to a zero mean and unit standard deviation.

In order to evaluate the robustness of our method to misspecification of the working structural models, the Gaussian‐distributed outcome Y had a mean specified in three ways: (a) as a function of the main terms $C_{0}$, $A_{0}$, $C_{1}$, and $A_{1}$, (b) the same as (a) but with an added interaction between $C_{0}$ and $C_{1}$ (covariate interaction) and c) the same as (a) but with an added interaction between $A_{0}$ and $C_{1}^{2}$ (effect modification). We specified the linear model for $q_{1}$ as containing only the main terms of all covariates; the linear model for $q_{0}$ also contained interactions between $C_{0}$ and $A_{0}$ and a squared term for $I_{0}$. The complete data‐generating mechanism is given in Web Appendix C.1.

We varied the sample sizes from n=200,500,1000. Table 1 reports $\sqrt{n}$ times the absolute bias and n times Monte Carlo mean squared error (MSE) over 1000 draws for each estimator for these three outcome‐generating models. The variable selection results for the proposed estimators are given in Table 2. In Scenario 1 (a) where the models used for the outcome process were correctly specified, the G‐computation estimator was unbiased with the lowest standard errors. All IPTW estimators were unbiased since they adjusted for all confounders; the oracle IPTWs had lower MSE than the IPTW adjusting for all covariates (“full IPTW”) though there was no difference between the two oracles. LOAL performed as well as the oracles, as did the fused LOAL. In Scenario 1 (b), all outcome models were misspecified and G‐Computation was the most biased. The full and oracle IPTW estimators were consistent but held some bias at these sample sizes; the oracle IPTW estimators had similar MSEs that were smaller than the full IPTW. LOAL and fused LOAL had lower MSE than the oracle IPTWs and comparable bias. In Scenario 1 (c), the G‐computation was highly biased but the full and oracle IPTW estimators were consistent with little bias. The oracles had the lowest MSE. The LOAL and fused LOAL were more biased than the oracle IPTWs and had higher MSE, but were still less biased than the G‐computation and had lower MSE than the full IPTW.

**TABLE 1:** Scenario 1: $\sqrt{n}$ times the absolute value of bias (n times mean squared error) of methods estimating the parameters in the MSM of Equation (1). IPTW oracle fits a glm of the target propensity score model with correctly selected variables and fused coefficients. The Fused LOAL uses the estimates of LOAL for the adaptive weights.

Method∖Scenario	(a) Outcome model with main terms			(b) Outcome model with covariate interaction			(c) Outcome model with effect modification		
	$\mu_{0}$	$\mu_{1}$	$\mu_{2}$	$\mu_{0}$	$\mu_{1}$	$\mu_{2}$	$\mu_{0}$	$\mu_{1}$	$\mu_{2}$
True values	−1.5	1.5	1.25	1	2.75	1.25	−1.5	4	5
*n = 200*									
G‐comp main terms	0.0 (37)	0.1 (24)	0.0 (21)	7.9 (183)	0.2 (185)	0.9 (110)	29.4 (461)	20.2 (362)	12.0 (232)
IPTW full main terms	0.3 (55)	0.4 (43)	0.2 (40)	5.7 (224)	0.1 (261)	0.2 (145)	1.9 (256)	1.8 (330)	3.4 (188)
IPTW oracle select	0.2 (46)	0.3 (36)	0.2 (33)	4.7 (202)	0.2 (229)	0.0 (135)	1.0 (186)	1.5 (259)	2.7 (152)
IPTW oracle select and fuse	0.1 (46)	0.4 (36)	0.1 (33)	4.7 (202)	0.1 (231)	0.1 (135)	0.9 (185)	0.9 (266)	2.7 (153)
LOAL	0.2 (46)	0.5 (37)	0.2 (34)	6.0 (190)	0.2 (209)	0.5 (123)	4.9 (228)	5.6 (284)	5.7 (166)
Fused LOAL	0.1 (46)	0.6 (36)	0.2 (34)	6.0 (191)	0.2 (213)	0.5 (123)	4.9 (228)	5.5 (285)	5.6 (166)
*n = 500*									
G‐comp main terms	0.1 (55)	0 (39)	0.1 (33)	11.8 (342)	0.8 (292)	2.1 (183)	45.5 (1061)	32.1 (798)	18.1 (473)
IPTW full main terms	0.5 (97)	0.4 (82)	0.4 (76)	6.1 (478)	0.1 (563)	0.3 (303)	3.0 (516)	4.2 (673)	3.9 (354)
IPTW oracle select	0.1 (77)	0.2 (67)	0.1 (60)	5.3 (395)	1.1 (464)	0.3 (280)	1.7 (352)	3.6 (503)	3.3 (283)
IPTW oracle select and fuse	0.1 (78)	0.2 (66)	0.1 (61)	5.3 (392)	0.9 (459)	0.3 (279)	1.7 (342)	3.2 (495)	3.3 (281)
LOAL	0.1 (80)	0.4 (63)	0.1 (63)	7.2 (375)	0.5 (432)	0.3 (255)	6.9 (381)	7.0 (537)	7.5 (304)
Fused LOAL	0.0 (81)	0.4 (63)	−0.1 (64)	7.3 (372)	0.4 (424)	0.3 (253)	6.9 (382)	6.8 (542)	7.4 (304)
*n = 1000*									
G‐comp main terms	0.1 (81)	0.0 (53)	0.0 (48)	16.6 (606)	1.5 (411)	2.9 (252)	63.9 (2063)	43.4 (1459)	25.5 (871)
IPTW full main terms	0.3 (148)	0.3 (121)	0.2 (117)	5.3 (741)	0.0 (1042)	0.2 (517)	3.2 (753)	2.6 (1081)	4.7 (546)
IPTW oracle select	0.1 (115)	0.4 (95)	0.1 (87)	5.3 (613)	0.9 (778)	0.8 (439)	1.1 (524)	2.1 (793)	3.7 (420)
IPTW oracle select and fuse	0.0 (116)	0.4 (94)	0.1 (88)	5.2 (612)	0.9 (784)	0.8 (439)	1.1 (524)	1.7 (810)	3.6 (424)
LOAL	0.1 (117)	0.4 (94)	0.1 (91)	6.7 (571)	0.2 (726)	0.2 (394)	8.1 (590)	11.1 (930)	8.9 (479)
Fused LOAL	0.1 (118)	0.4 (94)	0.0 (92)	6.8 (568)	0.2 (727)	0.2 (394)	8.2 (592)	10.8 (936)	8.8 (481)

**TABLE 2:** Scenario 1: Proportion selection of each covariate into each treatment model and fusion of the coefficients of common terms across the two models, out of 1000 runs. $C_{0}$ and $C_{1}$ are true confounders and $I_{0}$ and $I_{1}$ are both instruments. The coefficients for $C_{0}$ are common across the two models under the target adjustment set. The true βs are the coefficients in the working models corresponding to Equations (5) and (6). The $\lim_{n\to \infty }\hat{\beta }$ are the converging values of the estimates of β, which depend on the specification of the q models. Fused LOAL was implemented in two ways: first, taking initial values of $\hat{\alpha }$ as estimated in a variable selection oracle model, and second, taking initial values from the LOAL (the former procedure being independent of the outcome).

n	Method	Selected covariates							
		$A_{0}$ model		$A_{1}$ model				Fused nonzero coefficients	
		$C_{0}$	$I_{0}$	$C_{0}$	$I_{0}$	$C_{1}$	$I_{1}$	$C_{0}$	$I_{0}$
*Fusion results with oracle* $\hat{\alpha }$ *(independent of scenario)*									
**200**	Fused LASSO	1.00	0.00	1.00	0.00	1.00	0.00	0.99	0.00
**500**	Fused LASSO	1.00	0.00	1.00	0.00	1.00	0.00	0.99	0.00
**1000**	Fused LASSO	1.00	0.00	1.00	0.00	1.00	0.00	0.99	0.00
*(a) Outcome generating model with main terms*									
**200**	Fused LOAL	1.00	0.12	0.79	0.00	0.94	0.03	0.77	0.00
**500**	Fused LOAL	1.00	0.03	0.90	0.00	0.99	0.00	0.89	0.00
**1000**	Fused LOAL	1.00	0.01	0.95	0.00	1.00	0.00	0.94	0.00
	**True** β **s**	1.50	0.00	0.50	0.00	1.41	0.00		
	$\lim_{\text{n}\to \infty }\hat{\beta }$	1.50	0.00	0.50	0.00	1.65	0.00		
*(b) Outcome generating model with covariate interaction*									
**200**	Fused LOAL	0.96	0.09	0.79	0.03	0.56	0.17	0.75	0.00
**500**	Fused LOAL	1.00	0.08	0.93	0.02	0.71	0.12	0.84	0.00
**1000**	Fused LOAL	1.00	0.06	0.98	0.01	0.84	0.14	0.83	0.00
	**True** β **s**	2.75	0.00	1.75	0.00	1.41	0.00		
	$\lim_{\text{n}\to \infty }\hat{\beta }$	2.70	0.00	1.73	0.00	1.64	−0.04		
*(c) Outcome generating model with effect modification*									
**200**	Fused LOAL	1.00	0.16	0.24	0.03	1.00	0.21	0.22	0.00
**500**	Fused LOAL	1.00	0.13	0.32	0.02	1.00	0.17	0.28	0.00
**1000**	Fused LOAL	1.00	0.11	0.43	0.02	1.00	0.17	0.34	0.00
**5000**	Fused LOAL	1.00	0.06	0.78	0.01	1.00	0.16	0.60	0.00
	**True** β **s**	4.00	0.00	0.50	0.00	4.95	0.00		
	$\lim_{\text{n}\to \infty }\hat{\beta }$	5.40	0.04	0.61	0.05	7.82	0.09		

In Table 2, we give the proportion of each selected variable and fused coefficient for each of the proposed methods. At the top of the table, we see that, when the oracle estimates of α (i.e., estimated with correctly selected propensity score models) are used to weight the fused LASSO, the $L_{0}$ coefficients are correctly fused 99% of the time at all sample sizes. In Scenario 1 (a), LOAL correctly selected the covariates with nonzero β coefficients in the working structural models 79%−100% of the time for all sample sizes, with greater true positive rates for larger β values. The method also correctly omitted the instruments with almost no false positives by n=500. The success of fusion notably depended on the success of the variable selection, so that the $L_{0}$ covariates fused when the true model was selected in the first phase. In Scenarios 1 (b) and 1 (c) where the models to estimate $q_{t}$ were misspecified, the convergence of the covariate selection was slower. In Scenario 1 (c), the $\hat{\beta }$ did not converge to zero for the instruments, making it so that de‐selection of instruments was not consistent (though the selection of confounders was consistent, but with slow convergence). Since fused LASSO relies on the correct covariate selection, the proportion of fusion was lower than for the other scenarios.

### 5.2 Higher‐Dimensions, More Time‐Points, and Varied Instrument Strengths, Comparisons Across Estimators, and Inference

We additionally ran a higher dimensional scenario (Scenario 2), which demonstrated the good performance of the LOAL and Fused LOAL in terms of estimation, variable selection, and fusion, with 30 covariates and two time points. We ran a third scenario with five time‐points, demonstrating primarily that the smoothing by Fused LOAL approaches the oracle estimation and can positively impact estimation variance. Finally, for the first two scenarios, we compared the performance of LTMLE, implemented with and without LOAL, LTMLE with covariate screening and stacking (we used the superlearner R package including main terms logistic regressions, logistic regressions with main terms and interactions, and variable screeners, with all screening applied separately to the treatment and outcome models, respectively), and C‐LTMLE (a comparator variable selection method). While C‐LTMLE was slightly better than LOAL in terms of MSE when the outcome models were correctly specified, LTMLE with LOAL performed better when they were not. A major benefit of LOAL over C‐LTMLE is that it dramatically lowers computational complexity. LTMLE with variable screening unsurprisingly did not perform as well as the causal variable selection methods because variable screening applied directly to the treatment models will prioritize the selection of variables correlated with treatment, including instruments. See Web Appendix C.2–C.4 for details. In addition, to further investigate the potential for inference of the LOAL, we applied the m‐out‐of‐n bootstrap to Scenarios 1 (a) and 2. This bootstrap method is designed to improve bootstrap inference in settings where the standard bootstrap fails, including in covariate selection settings [41, 42, 43]. However, we observed undercoverage of the bootstrap‐based confidence intervals in our setting. Full details are provided in Web Appendix Section C.5. Finally, we compared the performance of LOAL versus the full model IPTW while firstly varying the strength of one instrument and secondly shifting all the propensity scores from lower to higher values. LOAL had far superior performance in terms of n times MSE across all scenarios.

## 6 Example: The Nicotine Dependence in Teens (NDIT) Study

We now illustrate the application of our proposed methodology using data from the Nicotine Dependence in Teens (NDIT) study to estimate the effect of the timing of alcohol initiation during adolescence on depressive symptoms in early adulthood. The NDIT study is a prospective longitudinal study initiated in 1999–2000, comprising 1294 grade seven students recruited from 10 high schools in Montréal, Canada [44]. Self‐report questionnaires were administered at three‐month intervals, resulting in a total of 20 cycles from 1999 to 2005. An additional post‐high school survey was conducted in 2007 or 2008. Data were collected from repeated assessments of a wide range of sociodemographic, substance use, psychosocial, lifestyle, and physical and mental health variables. We consider data from 1231 students who were in grade seven in September 1999 and who were not previous regular (at least weekly use) alcohol users.

The baseline variables (cycle 1) included in our analysis were reported sex (female vs. male), mother's education (less than university vs. at least some university), single‐parent home, French spoken at home, country of birth (outside Canada vs. Canada), self‐esteem, impulsivity, and novelty‐seeking. The time‐varying covariates L considered were current depressive symptoms, participation in team sports, family‐related stress, other type of stress, worry about weight, and ever smoked. The exposure $A_{t}$ was the indicator of initiation of regular alcohol use at or before cycle t. Note that if $A_{t}=1$ at a given time, then $A_{k}=1$ at all times k>t by our definition. We considered data from cycles 1 to 5, spanning calendar years 1999 to 2000, for the time‐varying covariates and exposure. The outcome Y was depressive symptoms experienced within the past two weeks as measured using the Major Depressive Inventory (MDI) in 2007 or 2008 [45] when participants were age 20.4 years on average (i.e., approximately two years after cycle 20). The outcome is a continuous score ranging from 0 to 50, with higher scores indicating more severe symptoms. Since not all participants initially recruited took part in all cycles of the study, we denote loss to follow‐up (i.e., censoring) by cycle t as $C_{t}=1$, and $C_{t}=0$ otherwise. The observed data structure is written as $O=(L_{1},A_{1},L_{2},C_{2},A_{2},\cdots \;,A_{5},L_{6},C_{6},Y).$ Note that $L_{1}$ contains the baseline covariates in addition to the time‐varying covariates at the first time.

We denote an arbitrary exposure pattern as $\bar{a}=(a_{1},\cdots \;,a_{5})$. Define 𝒟 as the treatment regimen space, corresponding to the 6 possible treatment patterns: initiation at time 1, 2, 3, 4, or 5, or no initiation at any time point. For example, initiation at time 2 is represented as (0,1,1,1,1). The parameters of interest were defined through the working MSM

(8) $$\begin{array}{ll} & 𝔼[Y^{\bar{a}}|\text{Sex}]=\mu_{0}+\mu_{1}\text{Sex}+\mu_{2}cum(\bar{a})+\mu_{3}\{\text{Sex}\times cum(\bar{a})\}, \end{array}$$

where $cum(\bar{a})$ gives the number of exposed time points in $\bar{a}$.

We extended our methods to incorporate the simultaneous presence of time‐dependent treatment and censoring. We considered the following implementations of IPTW and LTMLE:

- IPTW full: Fit stratified models for the probability of being exposed (and censored, respectively) at each given time according to all previous covariates' main terms among participants who were previously unexposed and uncensored.
- IPTW LOAL and IPTW fused LOAL: Included selected variables in the treatment and censoring models using the LOAL and fused LOAL procedures, respectively.
- LTMLE full: Included all covariates in the stratified treatment and censoring models.
- LTMLE LOAL and LTMLE fused LOAL: Included only the covariates selected using the LOAL and fused LOAL procedures, respectively, in the treatment and censoring models.
- LTMLE SL: LTMLE with superlearner screening for stratified treatment, censoring and outcome models. The library contains “SL.mean,” “SL.glm,” “SL.gam,” “SL.gam, screen.randomForest,” “SL.glm.interaction,” “SL.glm.interaction, screen.randomForest”, “SL.earth,” “SL.earth, screen.randomForest.”

For all implementations, the outcome models included the main terms of the baseline and time‐varying covariates, current and lagged exposure terms, and the first‐order interactions of sex and exposures for uncensored participants.

For LOAL and fused LOAL, we performed variable selection and fusion for the treatment model and censoring model separately, but the penalization parameters $\lambda_{n}^{a}$ (for treatment) and $\lambda_{n}^{c}$ (for censoring) were selected jointly by minimizing the sum of two longitudinal balancing metrics over covariates and times with respect to treatment and censoring at each time point. For fused LOAL, the penalty graph connected common baseline variables across time points, as well as common time‐varying variables with the same lag across time points (e.g., corresponding $L_{t-1}$ variables are connected together when modeling $A_{t}$, and when modeling $C_{t}$).

We fixed the tuning parameter γ at 2.5 and used 20 candidate values for the tuning parameters $\lambda^{a}$ with the range $\left(e^{-4},e^{8}\right)$ and $\lambda^{c}$ with the range $\left(e^{-5},e^{10}\right)$, with values increasing evenly on a log scale, and which, at the extremes, included both null and complete variable selection and fusing.

The full treatment model included 135 parameters (including five intercepts) and was reduced to 37 parameters by LOAL, where the variables sex and current depressive symptoms were selected in each time period. The fusion step further reduced the number of parameters to 23, a reduction of 83% in the number of parameters as compared to the full model (see Table 3). The full censoring model included 180 parameters (including five intercepts and 15 coefficients of past treatments), of which 112 remained after LOAL and 55 after fused LOAL, representing a reduction of 69% of the number of parameters. The variables selected in the censoring model included sex, country of birth, current depressive symptoms, ever smoked, family‐related stress, other stress, participation in team sports and worry about weight (see Table 4).

**TABLE 3:** Selected and fused parameters in the treatment model. “CurDep” represents current depressive symptoms; “FamStress” represents family stress; “TeamSport” represents participation in team sports; “WorWeight” represents worry about weight; “NA” represents that the time‐varying variable is not applicable at the given time. A blank space means that the variable was not selected by the LOAL in the first step. The values are color coded such that common colors indicate fused parameters.

Variable ∖ Time	1	2	3	4	5
Intercept	−4.067	−3.475	−3.898	−3.609	−1.469
Sex	−0.313	−0.313	−0.313	−0.313	−0.313
CountryBirth	−0.838	−0.838		−0.838	−0.838
MotherEducation					0.491
${\text{CurDep}}_{t=1}$	1.493				
${\text{CurDep}}_{t=2}$	NA	1.493			
${\text{CurDep}}_{t=3}$	NA	NA	1.493	0.127	
${\text{CurDep}}_{t=4}$	NA	NA	NA	1.493	0.127
${\text{CurDep}}_{t=5}$	NA	NA	NA	NA	1.493
${\text{EverSmoke}}_{t=3}$	NA	NA	1.349	1.455	0.916
${\text{FamStress}}_{t=2}$	NA				−0.208
${\text{FamStress}}_{t=4}$	NA	NA	NA		0.072
${\text{OtherStress}}_{t=5}$	NA	NA	NA	NA	0.081
${\text{TeamSport}}_{t=3}$	NA	NA		−0.020	0.147
${\text{TeamSport}}_{t=5}$	NA	NA	NA	NA	0.117
${\text{WorWeight}}_{t=1}$		0.277		0.410	0.074
${\text{WorWeight}}_{t=3}$	NA	NA	−0.119		
${\text{WorWeight}}_{t=4}$	NA	NA	NA	−0.119	0.277

**TABLE 4:** Selected and fused parameters in the censoring model. “CurDep” represents current depressive symptoms; “FamStress” represents family stress; “TeamSport” represents participation in team sports; “WorWeight” represents worry about weight; ‘NA’ represents that the time‐dependent variable is not applicable at the given time. A blank space means that the variable was not selected by the LOAL in the first step. The values are color coded according to fused clique.

Variable∖Time	2	3	4	5	6
Intercept	−4.980	−4.106	−2.445	−1.064	−1.443
A1	2.458			0.423	−0.810
A2	NA		−1.395	0.037	−1.429
A3	NA	NA	0.840	−0.594	3.187
A4	NA	NA	NA	0.709	−1.946
A5	NA	NA	NA	NA	−0.097
Sex	−0.336	−0.336	−0.336	−0.336	−0.336
SelfEsteem	−0.133	−0.133	−0.133	−0.133	
CountryBirth	0.760	0.760	0.760	0.760	0.760
MotherEducation			−0.255	−0.255	
${\text{CurDep}}_{t=1}$			−0.823	−0.360	−0.263
${\text{CurDep}}_{t=2}$	−0.242	−0.167	0.358		−0.360
${\text{CurDep}}_{t=3}$	NA	−0.242	−0.167	0.358	
${\text{CurDep}}_{t=4}$	NA	NA	−0.242	−0.167	0.358
${\text{CurDep}}_{t=5}$	NA	NA	NA	−0.242	−0.167
${\text{CurDep}}_{t=6}$	NA	NA	NA	NA	−0.242
${\text{EverSmoke}}_{t=1}$		0.235	0.264	−0.001	0.568
${\text{EverSmoke}}_{t=2}$		−0.192	0.235	0.264	−0.001
${\text{EverSmoke}}_{t=3}$	NA	0.506	−0.192	0.235	
${\text{EverSmoke}}_{t=4}$	NA	NA	0.506	‐0.192	
${\text{EverSmoke}}_{t=5}$	NA	NA	NA	0.506	
${\text{EverSmoke}}_{t=6}$	NA	NA	NA	NA	0.506
${\text{FamStress}}_{t=2}$	0.022	0.044	−0.039	0.329	−0.246
${\text{FamStress}}_{t=3}$	NA		0.044	−0.039	
${\text{FamStress}}_{t=4}$	NA	NA		0.044	−0.039
${\text{FamStress}}_{t=5}$	NA	NA	NA	0.022	0.044
${\text{FamStress}}_{t=6}$	NA	NA	NA	NA	0.022
${\text{OtherStress}}_{t=1}$	0.070	−0.219	0.353	0.150	−0.054
${\text{OtherStress}}_{t=3}$	NA			−0.219	
${\text{OtherStress}}_{t=4}$	NA	NA	−0.025	0.070	
${\text{OtherStress}}_{t=5}$	NA	NA	NA	−0.025	0.070
${\text{TeamSport}}_{t=2}$	−0.105	−0.185	0.128	0.004	0.191
${\text{TeamSport}}_{t=3}$	NA		−0.185	0.128	0.004
${\text{TeamSport}}_{t=4}$	NA	NA	−0.105	−0.185	
${\text{TeamSport}}_{t=5}$	NA	NA	NA	−0.105	−0.185
${\text{TeamSport}}_{t=6}$	NA	NA	NA	NA	−0.105
${\text{WorWeight}}_{t=1}$	−0.018	0.053	0.044	−0.388	0.012
${\text{WorWeight}}_{t=2}$	0.145				
${\text{WorWeight}}_{t=3}$	NA	0.145			
${\text{WorWeight}}_{t=4}$	NA	NA	0.145	−0.018	0.053
${\text{WorWeight}}_{t=5}$	NA	NA	NA	0.145	−0.018
${\text{WorWeight}}_{t=6}$	NA	NA	NA	NA	0.145

Estimated coefficients and standard errors are presented in Table 5. The standard errors of the four LTMLE estimates were obtained through the influence functions without accounting for variable selection and are thus not valid post‐inference standard errors. For IPTW, we applied the robust sandwich variance estimator. All methods consistently indicated that females had more severe depressive symptoms compared to males. Point estimates from IPTW full, LTMLE full, LTMLE SL, LTMLE LOAL, and LTMLE fused LOAL suggested that early alcohol initiation was associated with more depressive symptoms during young adulthood among male participants. All IPTW implementations suggested that alcohol initiation in female participants was associated with less severe depressive symptoms; however, the LTMLE implementations concluded null or harmful impacts of earlier drinking initiation for both sexes. Notably, the incorporation of propensity scores limited to covariates selected by LOAL dramatically reduced standard error estimates in both the IPTW and LTMLE analyses. In addition, LOAL plus fusion more than halved the estimated standard error of the LTMLE estimator compared to LTMLE with only LOAL. LOAL was able to limit the extreme cumulative probabilities of treatment and censoring used in the analysis; see Appendix Table D1.

**TABLE 5:** Estimates of the MSM parameters for the NDIT study application. Estimated standard errors are presented in brackets. IPTW [LTMLE] full represents IPTW [LTMLE] with pooled treatment models including all covariate main terms and pooled censoring models including all covariate main terms and treatment terms; IPTW [LTMLE] LOAL represents IPTW [LTMLE] with pooled treatment and censoring models after covariate selection by LOAL; IPTW [LTMLE] fused LOAL represents IPTW [LTMLE] with pooled treatment and censoring models with both selection by LOAL and coefficient fusion; LTMLE SL represents LTMLE with superlearner screening for treatment, censoring, and outcome models.

Method∖Coefficient	Intercept	Female sex	cum $\left(\;\bar{a}\;\right)$	$\text{Female sex}\times \text{cum}(\;\bar{a}\;)$
IPTW full	7.397 (1.119)	5.708 (2.177)	0.583 (0.646)	−1.833 (1.564)
IPTW LOAL	8.203 (0.975)	5.774 (1.898)	−0.224 (0.438)	−0.352 (0.844)
IPTW fused LOAL	8.553 (0.943)	4.223 (1.452)	−0.047 (0.636)	−0.455 (1.038)
LTMLE full	7.361 (0.337)	3.569 (0.573)	0.072 (0.253)	0.008 (0.504)
LTMLE LOAL	7.570 (0.104)	3.479 (0.182)	0.005 (0.085)	0.028 (0.154)
LTMLE fused LOAL	7.712 (0.028)	3.500 (0.045)	0.002 (0.042)	−0.008 (0.076)
LTMLE SL	7.571 (0.279)	3.477 (0.436)	0.064 (0.172)	0.005 (0.360)

Complete details of the application and results are given in Web Appendix D.

## 7 Discussion

Many causal confounder selection methods have been proposed for point treatment settings but very few for longitudinal data. Despite this, in practice, variable selection methods are highly utilized; a 2019 descriptive review found that 69/299 (24%) of articles published in epidemiology journals explicitly used data‐driven variable selection methods [46]. In this paper, we extended the Outcome Adaptive LASSO propensity score variable selection approach of Shortreed and Ertefaie [25] to the setting with time‐varying treatment over discrete time points. We first estimate regularized coefficients of the time‐saturated propensity score models. We then fuse the resulting nonzero coefficients using a generalized adaptive fused LASSO. Allowing for sparse model identification can avoid forcing a Markov‐type assumption where we assume a priori that treatment can only depend on the most recent values of the time‐updated covariates and baseline covariates. Oracle properties of the outcome‐adaptive LASSO [25] and the generalized adaptive fused LASSO [32] guarantee oracle performance of these estimators in larger samples. In our setting, this means that the fused LOAL will select then fuse the coefficients correctly according to the marginal pooled treatment model as sample size increases.

Our simulation studies show that implementation of our method can improve estimation by IPTW and LTMLE compared to the same estimators without variable selection. The success of the selection relied on the specification of the outcome model to the extent that the estimates of the βs in the working model converged correctly to either zero or nonzero; however, the success of variable selection also depended on the variance of the estimators of these βs. The fusion was highly successful whenever the correct covariates were selected in the LOAL step.

The application demonstrated the usage of our method in a realistically complex longitudinal study in epidemiology where the interest was in estimating the effect of the initiation time of regular alcohol consumption on depression symptoms in young adulthood. We extended our method to select adjustment variables in both the treatment and censoring models, and used the balance criterion to jointly select the tuning parameter for the two models. The reduction of covariates and fusion of coefficients in the treatment and censoring models both led to apparent major gains in efficiency.

An important limitation of all frequentist covariate selection methods for the propensity score that exclude instruments is that uniformly valid inference is not available [47, 48]. This means that in practice, it may be best to avoid covariate selection in combination with IPTW and LTMLE if possible. LOAL and fused LOAL may still be used to identify the relevant low‐dimensional set of confounding variables needed and the appropriateness of pooling over time for analyses on a separate observational dataset. However, C‐TMLE has been shown to be asymptotically linear under certain conditions [8], relying on cross‐validation to select the optimal number of selection steps. While we do not expect this version of LOAL implemented with IPTW to be asymptotically linear, doubly robust inference approaches that also target residual bias terms due to incorrectly estimated treatment functions are a potential avenue for valid inference [49, 50]. Bootstrap methods valid under very general conditions, such as m‐out‐of‐n and multiplier bootstrap, are other possibilities [41, 51]. Another limitation of our method, as currently proposed with working parametric models, is that it cannot handle nonlinearities or interactions between covariates in the propensity score models. Potential extensions of our method may incorporate nonparametric approaches used in the single time‐point setting such as causal ball screening [21] for high‐dimensional covariates and outcome highly adaptive lasso [50], the latter of which has valid closed‐form expressions for confidence intervals. To conclude, we consider this work as a step forward in the development of nonparametric shrinkage estimators that trade‐off bias and variance in the MSM parameter estimation for longitudinal treatments while allowing for valid inference.

## Funding

This work was supported by the Natural Sciences and Engineering Research Council of Canada (Grant No. RGPIN‐2021‐03019), Canada Research Chairs (Grant No. CRC‐2018‐00228), the National Institutes of Health (Grant Nos. R33NS120240, R01ES034021, R01DA048764), and the Fonds de recherche du Québec (Grant No. 312198).

## Conflicts of Interest

The authors declare no conflicts of interest.

## Supporting information


**Data S1**: The R codes used for the simulations are available at https://github.com/Schnitzer‐Biostats‐Lab/Longitudinal‐outcome‐adaptive‐LASSO.

## Data Availability

NDIT data are available upon request. Access to NDIT data is open to any university‐appointed or affiliated investigator upon successful completion of the application process. Master's, doctoral, and postdoctoral students may apply through their primary supervisor. To gain access, applicants must complete a data access form available on the NDIT website (https://www.CELPHIE.ca) and return it to the principal investigator (jennifer.oloughlin@umontreal.ca). The procedure to obtain access to NDIT data is described in O'Loughlin, J., Dugas, E. N., Brunet, J., DiFranza, J., Engert, J. C., Gervais, A., Gray‐Donald, K., Karp, I., Low, N. C., Sabiston, C., Sylvestre, M. P., Tyndale, R. F., Auger, N., Belanger, M., Barnett, T., Chaiton, M., Chenoweth, M. J., Constantin, E., Contreras, G., Kakinami, L., Labbe, A., Maximova, K., McMillan, E., O'Loughlin, E. K., Pabayo, R., Roy‐Gagnon, M. H., Tremblay, M., Wellman, R. J., Hulst, A., Paradis, G., 2015. Cohort Profile: The Nicotine Dependence in Teens (NDIT) Study. Int J Epidemiol. 44 (5), 1537–1546. doi: https://doi.org/10.1093/ije/dyu135.
