# Supplementary material for: Adaptive Sparsening and Smoothing of the Treatment Model for Longitudinal Causal Inference Using Outcome‐Adaptive LASSO and Marginal Fused LASSO
Source: Stat Med. 2026 Jan 22;45(1-2):e70316. doi: 10.1002/sim.70316 (PMC12826353; doi:10.1002/sim.70316)
Supplement: Supplementary file 1 — Data S1: The R codes used for the simulations are available at https://github.com/Schnitzer‐Biostats‐Lab/Longitudinal‐outcome‐adaptive‐LASSO. [file SIM-45-0-s001.pdf]

□

APPENDIX

ADAPTIVE SPARSENING AND SMOOTHING OF THE TREATMENT MODEL FOR LONGITUDINAL CAUSAL INFERENCE USING OUTCOME-ADAPTIVE LASSO AND MARGINAL FUSED LASSO

Mireille E Schnitzer, Denis Talbot, Yan Liu, David Berger, Guanbo Wang, Jennifer O'Loughlin, Marie-Pierre Sylvestre, and Ashkan Ertefaie

A BALANCE METRIC FOR VARIABLE SELECTION

Our tuning parameter selection for the LOAL evaluates the balance of every covariate or function of covariates at each time point between units with different current treatment values. It is similar to a summary of the longitudinal balancing metric in[1] (Diagnostic 3). However, unlike,[1] we do not subset on treatment history as this will result in vanishing data support for greater numbers of time points; our definition of the weights in the main manuscript, which does not stabilize conditional on past treatments, produces independence between past treatment and current covariates.[2] Each covariate $L_{t,k}$ considered at each time point $\tau \geq t$ will be weighted by the corresponding structural model coefficient ($\hat{\beta}_{\tau,t,k}$) divided by its standard error ($\sigma_{\hat{\beta}_{\tau,t,k}}$). We let $\hat{\boldsymbol{\alpha}}^{refit}(\lambda_n)$ represent the estimated values of the $\boldsymbol{\alpha}$ parameters using a logistic regression on the covariates selected by LOAL under tuning parameter value λ_n, where the value is defined as zero if the corresponding coefficient was not selected. Then, define the LOAL-weight for subject i at time τ as $\hat{w}^{\lambda_n}_{\tau,i}(\bar{a}_\tau) = w_\tau\{\bar{a}_\tau, \bar{\boldsymbol{l}}_{\tau,i}; \hat{\boldsymbol{\alpha}}^{refit}(\lambda_n)\}$ corresponding to the weights defined in the main manuscript. For the simple example, we use the metric

$$\sum_{k=1}^{p_0} \frac{|\hat{\beta}_{0,0,k}|}{\sigma_{\hat{\beta}_{0,0,k}}} \left| \frac{\sum_{i=1}^n a_{0,i} l_{0,k,i} \hat{w}^{\lambda_n}_{0,i}(1)}{\sum_{i=1}^n a_{0,i} \hat{w}^{\lambda_n}_{0,i}(1)} - \frac{\sum_{i=1}^n (1-a_{0,i}) l_{0,k,i} \hat{w}^{\lambda_n}_{0,i}(0)}{\sum_{i=1}^n (1-a_{0,i}) \hat{w}^{\lambda_n}_{0,i}(0)} \right|$$
$$+ \sum_{k=1}^{p_0} \frac{|\hat{\beta}_{1,0,k}|}{\sigma_{\hat{\beta}_{1,0,k}}} \left| \frac{\sum_{i=1}^n a_{1,i} l_{0,k,i} \hat{w}^{\lambda_n}_{1,i}(a_{0,i},1)}{\sum_{i=1}^n a_{1,i} \hat{w}^{\lambda_n}_{1,i}(a_{0,i},1)} - \frac{\sum_{i=1}^n (1-a_{1,i}) l_{0,k,i} \hat{w}^{\lambda_n}_{1,i}(a_{0,i},0)}{\sum_{i=1}^n (1-a_{1,i}) \hat{w}^{\lambda_n}_{1,i}(a_{0,i},0)} \right|$$
$$+ \sum_{k=1}^{p_1} \frac{|\hat{\beta}_{1,1,k}|}{\sigma_{\hat{\beta}_{1,1,k}}} \left| \frac{\sum_{i=1}^n a_{1,i} l_{1,k,i} \hat{w}^{\lambda_n}_{1,i}(a_{0,i},1)}{\sum_{i=1}^n a_{1,i} \hat{w}^{\lambda_n}_{1,i}(a_{0,i},1)} - \frac{\sum_{i=1}^n (1-a_{1,i}) l_{1,k,i} \hat{w}^{\lambda_n}_{1,i}(a_{0,i},0)}{\sum_{i=1}^n (1-a_{1,i}) \hat{w}^{\lambda_n}_{1,i}(a_{0,i},0)} \right|.$$

B ASYMPTOTICS OF THE GENERALIZED ADAPTIVE FUSED LASSO

Here we state a theorem on the convergence of the estimates of the generalized adaptive fused LASSO that does not include a sparsity-inducing penalty and is performed after variable selection. The result is directly connected to the main result in Viallon et al.[3] We present this statement with the purpose of giving details about the theoretical properties of the second stage of our procedure.

Recall that the parameter $\boldsymbol{\alpha} = (\boldsymbol{\alpha}_{0,-1}, \boldsymbol{\alpha}_{0,0}, \boldsymbol{\alpha}_{1,-1}, \boldsymbol{\alpha}_{1,0}, \boldsymbol{\alpha}_{1,1}, \boldsymbol{\alpha}_{1,2})$ is defined according to the model (4) in the main manuscript. Define $\boldsymbol{\alpha}^\dagger = (\alpha_{0,-1}, \boldsymbol{\alpha}^\dagger_{0,0}, \alpha_{1,-1}, \boldsymbol{\alpha}^\dagger_{1,0}, \boldsymbol{\alpha}^\dagger_{1,1}, \alpha_{1,2})$, a parameter vector potentially containing fixed zeros among the elements of $\boldsymbol{\alpha}^\dagger_{0,0}$, $\boldsymbol{\alpha}^\dagger_{1,0}$, and $\boldsymbol{\alpha}^\dagger_{1,1}$. The elements that are not fixed zeros are free parameters. Suppose that the marginalized distribution of the treatments $A_t; t = 0, 1$ corresponds to Bernoulli distributions with probability of success $m_\tau(\bar{L}_1, A_0; \boldsymbol{\alpha}^\dagger_0)$ where $\boldsymbol{\alpha}^{\dagger,*}$ are defined as the true parameter values under maximum likelihood. More specifically, the distribution of A_0 conditional on the elements of L_0 corresponding to non-fixed-zero components of $\boldsymbol{\alpha}^\dagger_{0,1}$ is Bernoulli with probability of success $m_0(\bar{L}_1, A_0; \boldsymbol{\alpha}^{\dagger,*})$; the distribution of A_1 conditional on A_0 and the elements of (L_0, L_1) corresponding to non-fixed-zero components of $(\boldsymbol{\alpha}^\dagger_{1,0}, \boldsymbol{\alpha}^\dagger_{1,1})$ is Bernoulli with probability of success $m_1(\bar{L}_1, A_0; \boldsymbol{\alpha}^{\dagger,*})$.

Let $\mathcal{J}^\dagger_{0,1}$ and $\mathcal{J}^\dagger_{1,1}$ denote the indices of the covariates corresponding to the non-zero elements of $\boldsymbol{\alpha}^\dagger_{0,0}$ and $\boldsymbol{\alpha}^\dagger_{1,0}$, respectively. We define a graph $\mathcal{G} = (V, E)$ with vertices $V = \{(0, 0, k_0), (1, 0, k_1); k_0 \in \mathcal{J}^\dagger_{0,0}$ and $k_1 \in \mathcal{J}^\dagger_{1,0}\}$ and edges E that connect all (pairs of) corresponding indices for $k \in \mathcal{J}^\dagger_{0,0} \cap \mathcal{J}^\dagger_{1,0}$. This is the graph that will be used to run the adaptive fused LASSO.

TABLE C1 Simulation Scenario 1 data generating mechanism

Variable	Generating Mechanism
C_0	$\sim N(\text{mean} = 0, \text{sd} = 1)$
I_0	$\sim N(\text{mean} = 0, \text{sd} = 1)$
A_0	$\sim \text{Bernoulli}(\text{logit}(p) = 1.515C_0 + I_0)$
C_1	$\sim N(\text{mean} = C_0 + A_0, \text{sd} = 1)$
I_1	$\sim N(\text{mean} = C_0, \text{sd} = 1)$
A_1	$\sim \text{Bernoulli}(\text{logit}(p) = -0.5 + 0.5C_0 + 0.25C_1 + 0.5A_0 + I_1)$
Y for Scenario 1(a)	$\sim N(\text{mean} = -1.5 + 0.5C_0 + 0.5A_0 + C_1 + A_1, \text{sd} = 0.5)$
Y for Scenario 1(b)	$\sim N(\text{mean} = -1.5 + 0.5C_0 + 0.5A_0 + C_1 + A_1 + 2.5C_0C_1, \text{sd} = 0.5)$
Y for Scenario 1(c)	$\sim N(\text{mean} = -1.5 + 0.5C_0 + 0.5A_0 + C_1 + A_1 + 2.5A_0C_1^2, \text{sd} = 0.5)$

Define the estimator $\hat{\boldsymbol{\alpha}}^\dagger$ as the minimizer of

$$\sum_{\tau=0}^{1}\sum_{i=1}^{n}\left[a_{\tau,i}\log\{m_\tau(\bar{l}_{1,i}, a_{0,i}; \boldsymbol{\alpha}^\dagger)\} + (1-a_{\tau,i})\log\{1-m_\tau(\bar{l}_{1,i}, a_{0,i}; \boldsymbol{\alpha}^\dagger)\}\right] + \lambda_{1,n}\sum_{k\in\mathcal{J}_{0,0}^\dagger\cap\mathcal{J}_{1,0}^\dagger,}\frac{|\alpha_{1,0,k}^\dagger - \alpha_{0,0,k}^\dagger|}{|\tilde{\alpha}_{1,0,k}^\dagger - \tilde{\alpha}_{0,0,k}^\dagger|^{\gamma_1}} \tag{B1}$$

where $\tilde{\alpha}_{1,0,k}^\dagger$ and $\tilde{\alpha}_{0,0,k}^\dagger$ are $\sqrt{n}$-consistent estimates of $\alpha_{1,0,k}^{\dagger,*}$ and $\alpha_{0,0,k}^{\dagger,*}$, respectively.

Now following[3] with adaptations to our setting, we define $\mathcal{J}^* = \{k \in \mathcal{J}_{0,1}^\dagger \cap \mathcal{J}_{1,1}^\dagger : \alpha_{0,1,k}^{\dagger,*} = \alpha_{1,1,k}^{\dagger,*}\}$, i.e. the set of indices where fusing should occur. Furthermore, let $\mathcal{B} = \{(0,1,k),(1,1,k) \in E : j \in \mathcal{J}^*\} \subseteq E$, which is a set of connected indices where the true values of the parameters are equal. Define the graph $\mathcal{G}_\mathcal{B} = (V, \mathcal{B})$ as the one containing the complete set of vertices of $\mathcal{G}$ but with edges only between the connected vertices of E where the corresponding parameters values are equal. Define s_0 as the number of connected components of the graph $\mathcal{G}_\mathcal{B}$.

Now define $\alpha_\mathcal{B}^* = (\alpha_{0,0}^*, \alpha_{0,1}^{\dagger,*}, \alpha_{1,0}^*, \alpha_{1,1,\mathcal{J}_{1,1}\setminus\mathcal{J}^*}^*, \alpha_{1,2}^{\dagger,*}, \alpha_{1,3}^*)^T$, which are the same as $\alpha^{\dagger,*}$ after removal of the redundant terms in $\alpha_{1,1}$ that are equal to their connected terms in $\alpha_{0,1}$; and let $\hat{\alpha}_\mathcal{B}$ be its estimate by the adaptive fused LASSO in (B1). Finally, define $\mathcal{B}_n = \{(0,1,k),(1,1,k) \in E : \hat{\alpha}_{0,1,k}^\dagger = \hat{\alpha}_{1,1,k}^\dagger\}$, the edges that fused in the procedure.

Theorem 1. *If $\lambda_{1,n}/\sqrt{n} \to 0$ and $\lambda_{1,n}n^{(\gamma_1-1)/2} \to \infty$, then under mild assumptions, the minimizer of (B1) satisfies $P(\mathcal{B}_n = \mathcal{B}) \to 1$ as $n \to \infty$ and $\sqrt{n}\left(\hat{\alpha}_\mathcal{B} - \alpha_\mathcal{B}^*\right)$ converges in distribution to a Gaussian distribution of dimension $s_0 + 3$ with mean zero.*

The mild assumptions are given explicitly in Viallon et al.[4] as AL1 and AL2. The proof of our theorem follows exactly the steps of their proof of Theorem 2 excluding the sparsity element.

C SIMULATION STUDY DETAILS AND EXTENDED RESULTS

C.1 Scenario 1 data generating mechanisms

The data in Scenario 1 were generated according to the left-hand DAG in Figure C1 and more specifically from the mechanisms presented in Table C1.

C.2 Scenario 2: higher dimensional covariates with two time-points

In this scenario, we generated 20 independent covariates at time 0 and 10 covariates at time 1, two treatments and a continuous outcome, according to the right-hand DAG in Figure C1 and more specifically the complete data generating mechanism in Table C2. At time 0 there were two confounders, jointly denoted C_0; two pure causes of the outcome, P_0; two instruments, I_0, and 14 spurious covariates S_0. At time 1 there were two confounders, C_1; two pure causes of the outcome, P_1, two instruments, I_1, and four spurious covariates S_1. All of the time 1 covariates were affected by the corresponding covariate at time 0 and also by the previous treatment A_0. The data were generated in such a way that the coefficients of both confounders in C_0 at the two time-points were equal in the marginal pooled treatment model that excluded instruments and spurious covariates. The coefficients of the two variables P_0 were equal to zero in this same model. The outcome was Gaussian with mean linear in the

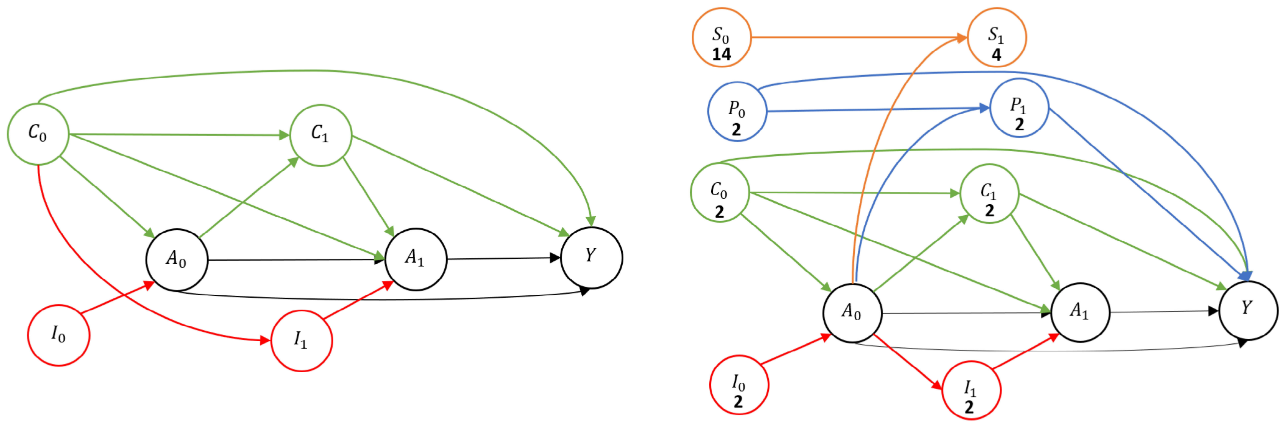

FIGURE C1 DAGs representing the data generation in Scenarios 1 (left) and 2 (right). The target variable selection retained all covariates labeled C and P (the latter only for Scenario 2 in both models).

TABLE C2 Simulation Scenario 2 data generating mechanism

Variable	Generating Mechanism
$C_{0,j}$ for $j = (1, 2)$	$\sim N(\text{mean} = 0, \text{sd} = 1)$
$P_{0,j}$ for $j = (1, 2)$	$\sim N(\text{mean} = 0, \text{sd} = 1)$
$I_{0,j}$ for $j = (1, 2)$	$\sim N(\text{mean} = 0, \text{sd} = 1)$
$S_{0,j}$ for $j = (1, ..., 14)$	$\sim N(\text{mean} = 0, \text{sd} = 1)$
A_0	$\sim \text{Bernoulli}(\text{logit}(p) = C_{0,1} + C_{0,2} + I_{0,1} + I_{0,2})$
$C_{1,1}$	$\sim N(\text{mean} = 0.5C_0 + 0.5A_0, \text{sd} = 1)$
$C_{1,2}$	$\sim N(\text{mean} = 0.2C_0 - A_0, \text{sd} = 1)$
$P_{1,1}$	$\sim N(\text{mean} = 0.5C_0 + 0.5A_0, \text{sd} = 1)$
$P_{1,2}$	$\sim N(\text{mean} = 0.2C_0 - A_0, \text{sd} = 1)$
$I_{1,1}$	$\sim N(\text{mean} = -0.5A_0, \text{sd} = 1)$
$I_{1,2}$	$\sim N(\text{mean} = A_0, \text{sd} = 1)$
$S_{1,j}$ for $j = (1, ..., 4)$	$\sim N(\text{mean} = 0.5C_0 + 0.2A_0, \text{sd} = 1)$
A_1	$\sim \text{Bernoulli}(\text{logit}(p) = 1.026C_{0,1} + 0.987C_{0,2} + 0.5A_0 + C_{1,1} + C_{1,2} + I_{1,1} + I_{1,2})$
Y	$\sim N(\text{mean} = 1 + 0.6C_{0,1} + 0.6C_{0,2} + 0.6P_{0,1} + 0.6P_{0,2} + 0.6C_{1,1} + 0.6C_{1,2}$ $+0.6P_{1,1} + 0.6P_{1,2} + 0.5A_0 + A_1, \text{sd} = 1)$

main terms of C, P, A_0, and A_1. The model to estimate q_1 conditioned on all main terms (and thus contained the truth) for all methods; the models for q_0 were linear in the main terms.

The $\sqrt{n}$-bias and n-MSE are given in the first three data columns of Table C4. G-computation was unbiased with the lowest MSE as it was approximately correctly specified. The oracle IPTWs produced lower bias and MSE than the full IPTW. While the LOAL and fused LOAL had higher bias than their oracle counterparts, they had lower MSE. Figure C2 gives the proportion selection for each covariate at each time point and proportion fused (and non-zero) for corresponding baseline covariates between the two time points. While the confounders C were selected nearly 100% of the time at all sample sizes, the selection of P varied between roughly 75-100%, and appeared to be slowly converging. The selection of instruments I varied between 10-20% and appeared to be slowly converging to zero. Spurious covariates S were selected less often (below 10%) and converged close to zero by $n = 1000$. The fusion of both C_0 variables quickly converged to almost 100% by $n = 1000$. The terms P_0 often fused when they were both selected and non-zero, about 60-75% of the time.

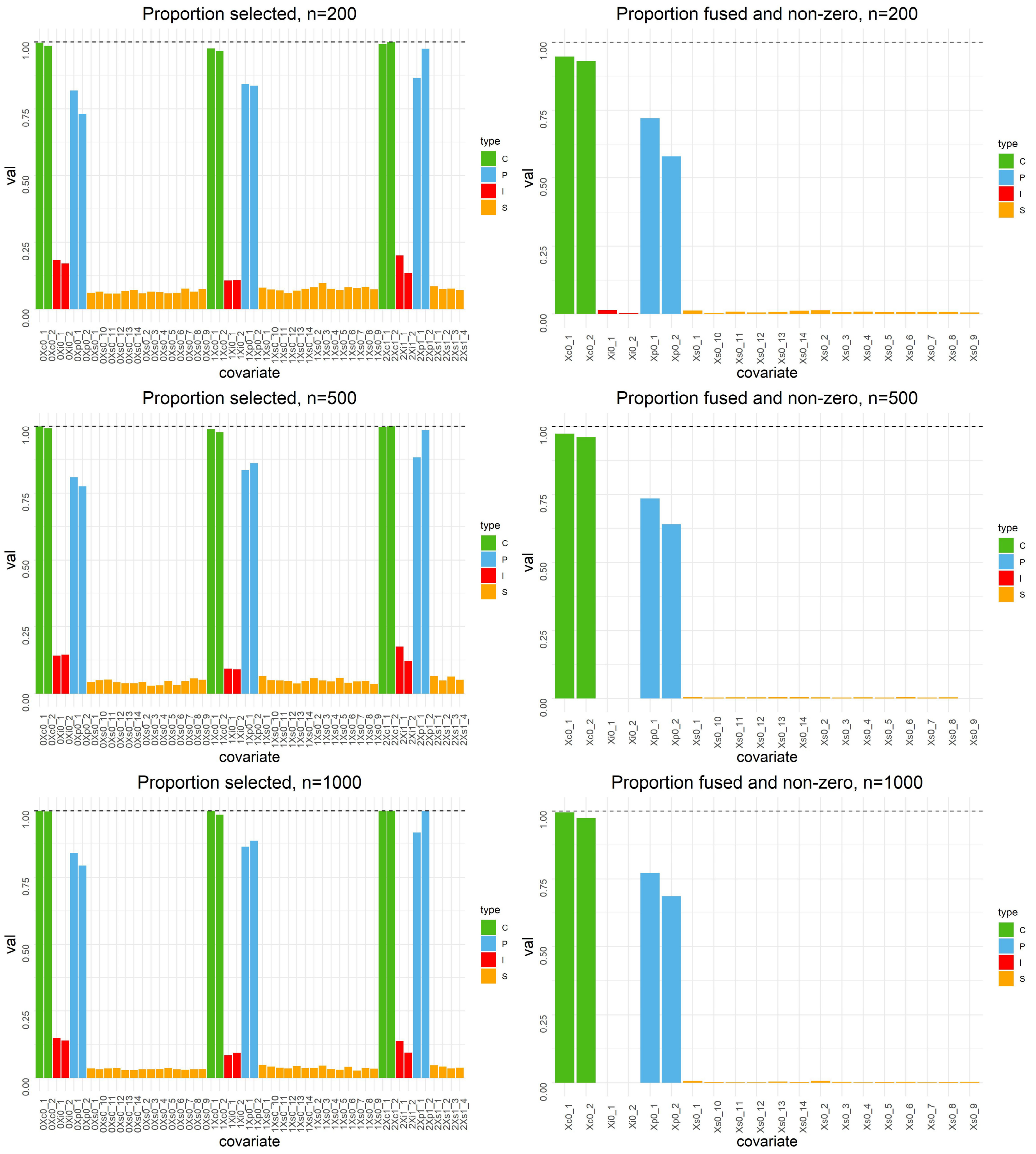

FIGURE C2 Proportion variable selection (left) and fusion (right) for $n = 200$ (top), $n = 500$ (middle), and $n = 1000$ (bottom) in Scenario 2. Labels: C are the confounders (should be selected), P are the pure causes of the outcome (should be selected), I are the instruments, and S are noise variables.

C.3 Scenario 3: five time-points with only baseline covariates

Finally, in order to demonstrate the potential usefulness of fusing, we developed a scenario with five treatments over time. Using the same approach as in Scenario 2, we simulated 20 baseline covariates (two confounders C_0, two pure predictors of

TABLE C3 Simulation Scenario 3 data generating mechanism

Variable	Generating Mechanism
$C_{0,j}$ for $j = (1, 2)$<br>$P_{0,j}$ for $j = (1, 2)$<br>$I_{0,j}$ for $j = (1, 2)$<br>$S_{0,j}$ for $j = (1, ..., 14)$	$\sim$ Multivariate normal distribution with mean 0 and the covariance matrix $\Sigma_{(20\times 20)} = \begin{bmatrix} 0.64 & 0.192 & 0.192 & \cdots & 0.192 \\ 0.192 & 0.64 & 0.192 & \cdots & 0.192 \\ \vdots & \vdots & \vdots & \vdots & \vdots \\ 0.192 & 0.192 & 0.192 & \cdots & 0.64 \end{bmatrix}$
A_0	$\sim$ Bernoulli(logit(p) = $0.5C_{0,1} + C_{0,2} - 0.5I_{0,1} - 0.5I_{0,2}$)
A_1	$\sim$ Bernoulli(logit(p) = $0.542C_{0,1} + 1.075C_{0,2} - 0.545I_{0,1} - 0.545I_{0,2} - 0.5A_0$)
A_2	$\sim$ Bernoulli(logit(p) = $0.568C_{0,1} + 1.142C_{0,2} - 0.565I_{0,1} - 0.569I_{0,2} - 0.5A_1$)
A_3	$\sim$ Bernoulli(logit(p) = $0.615C_{0,1} + 1.23C_{0,2} - 0.61I_{0,1} - 0.61I_{0,2} - 0.5A_2$)
A_4	$\sim$ Bernoulli(logit(p) = $0.66C_{0,1} + 1.322C_{0,2} - 0.655I_{0,1} - 0.655I_{0,2} - 0.5A_3$)
Y	$\sim N$(mean = $0.6C_{0,1} + 0.6C_{0,2} + 0.6P_{0,1} + 0.6P_{0,2} + 0.5A_0 + 0.5A_1 + 0.5A_2$ $+0.5A_3 + 0.5A_4$, sd = 1)

the outcome P_0, two instruments I_0, and 14 spurious covariates S_0). To facilitate the construction of this scenario, we did not generate covariates past the baseline. The data generation was done in such a way that, in the marginal pooled treatment model, all 5 of the coefficients for each confounder were equal, and all 10 of the outcome predictor coefficients were equal to zero. Thus, variable selection should reduce the number of parameters (including 5 intercepts and 10 coefficients of treatment) from 115 to 35, and fusion should further reduce the number of parameters to 19. See Table C3 for the complete data generating mechanism.

The $\sqrt{n}$-bias and n-MSE results are given in the last three columns of Table C4. G-computation was approximately correctly specified and unbiased. The full IPTW was more biased. IPTW with oracle variable selection decreased the bias and MSE relative to the full IPTW. IPTW with oracle variable selection and fusing produced smaller MSE compared to IPTW with oracle variable selection. Fused LOAL produced reductions in bias for μ_1 and MSE reductions for μ_0 and μ_1 compared to LOAL.

The selection and fusion results are given in Figure C3. The confounder variable selection was again close to ideal. The selection of pure causes of the outcome increased to above 90% as n increased. Instrument and spurious covariate selection was again low. We also verified that the fusion of non-zero coefficients of the confounders matched the selection of confounders and was near perfect for $n = 1000$.

C.4 Comparison with C-LTMLE and LTMLE with Super Learner with variable screening

We used the simulation Scenarios 1 and 2 to contrast the estimation of $\mathbb{E}(Y^{1,1})$ by LTMLE using variable selection with LOAL to variable selection with SL screening methods and to an implementation of C-LTMLE. The C-LTMLE was implemented for two time-points to estimate the interventional mean in previous work.[5] This C-LTMLE procedure uses initial estimates of $q_t; t = 1, 0$ to select covariates into the two propensity score models in a greedy fashion, creating a sequence of nested models with corresponding TMLE-updated estimates of $q_t; t = 0, 1$ with uniformly decreasing risk. Cross-validation is then used to select the point at which to stop, and this number of selections is used to determine the chosen propensity score models. (See[6] and[5] for more details.) Using the same linear main-terms models to estimate q_t throughout, we fit 1) LTMLE using propensity scores conditional on the full set of covariates, 2) LTMLE using Super Learner with library "SL.mean", "SL.glm", "SL.glm, screen.glmnet", "SL.glm.interaction", "SL.glm.interaction, screen.glmnet", 3) C-LTMLE, and 4) LTMLE using propensity scores with the covariates selected by LOAL.

The results in Table C5 show that both C-LTMLE and LTMLE with LOAL generally improved the bias and MSE over LTMLE implemented with all covariates or with superlearner. In Scenarios 1(a) and 2 when the outcome models were nearly correctly specified, both C-LTMLE and LTMLE with LOAL generally performed similarly in terms of bias, while C-LTMLE had lower MSE in higher-dimensional Scenario 2. However, with incorrect model specifications (Scenarios 1(b) and 1(c)), LTMLE LOAL had less bias and MSE than all other approaches compared here. Compared to LTMLE with LOAL, C-LTMLE was substantially slower, while LTMLE with superlearner screening had a similar runtime; for example, in Scenario 2 with $n = 1000$, C-LTMLE had a 90 second runtime while LTMLE with superlearner with variable screening took 9 seconds, and LTMLE with LOAL took only 3 seconds. However, the C-LTMLE procedure may be made more scalable with a preliminary ordering of the variables rather than the greedy procedure we employed.[7]

TABLE C4 Scenarios 2 and 3 $\sqrt{n}$ times the absolute value of bias (n times mean squared error) of methods estimating the parameters in the marginal structural model of equation (see Equation 1 in Section 2.1 of the manuscript). The Fused LOAL uses the estimates of LOAL for the adaptive weights.

Method\Scenario	Scenario 2, two time-points, $dim(L_0)$ = 20, $dim(L_1)$ = 10			Scenario 3, five time-points, $dim(L_0)$ = 20		
	μ_0	μ_1	μ_2	μ_0	μ_1	μ_2
True values	1.00	0.88	0.45	0.0	1.14	0.5
**n=200**						
G-comp main terms	0.3(11)	0.2(5)	0.4(7)	0.1(9)	0.1(4)	0.0(1)
IPTW full main terms	3.5(118)	1.5(40)	3.7(65)	2.1(54)	0.4(16)	0.8(8)
IPTW oracle select	2.2(41)	1.6(18)	2.0(27)	0.6(22)	0.2(8)	0.2(3)
IPTW oracle select and fuse	2.2(40)	1.5(17)	2.0(27)	0.6(20)	0.1(7)	0.2(3)
LOAL	2.8(36)	1.8(17)	2.6(25)	1.1(23)	0.4(8)	0.4(3)
Fused LOAL	2.9(36)	1.7(16)	2.6(25)	1(22)	0.3(7)	0.4(3)
**n=500**						
G-comp main terms	0.6(9)	0.2(5)	0.8(6.5)	0.3(8)	0(3)	0.1(1)
IPTW full main terms	4.0(153)	1.7(51)	3.9(90)	2.1(73)	0.7(18)	0.9(11)
IPTW oracle select	2.1(59)	1.4(23)	2.1(41)	0.6(23)	0.3(9)	0.3(4)
IPTW oracle select and fuse	2.1(59)	1.4(23)	2.1(41)	0.6(22)	0.3(8)	0.3(3)
LOAL	3.1(45)	2.0(20)	3.0(30)	1.1(25)	0.6(9)	0.5(4)
Fused LOAL	3.2(46)	1.9(20)	3.1(31)	1(24)	0.4(9)	0.5(4)
**n=1000**						
G-comp main terms	0.7(9)	0.3(5)	0.8(7)	0.1(8)	0(3)	0(1)
IPTW full main terms	4.4(182)	2.5(70)	4.3(114)	1.5(80)	0.6(20)	0.7(13)
IPTW oracle select	2.8(75)	2.0(30)	2.4(48)	0.3(28)	0.3(10)	0.2(4)
IPTW oracle select and fuse	2.8(75)	2.0(30)	2.4(48)	0.3(26)	0.3(9)	0.2(4)
LOAL	3.7(59)	2.5(26)	3.3(38)	0.8(30)	0.5(10)	0.4(5)
Fused LOAL	3.7(59)	2.5(26)	3.3(39)	0.8(29)	0.3(9)	0.4(5)

C.5 Inference using the m-out-of-n bootstrap

The m-out-of-n bootstrap is a resampling method designed to improve inference in settings where the standard bootstrap fails, especially when the parameter of interest is non-smooth, such as when it involves an extrema.[8–10] Instead of resampling n observations from a dataset of size n, this method draws only m observations with replacement where it requires that $m/n \to 0$ and $m \to \infty$.[8] A key challenge is choosing an appropriate m: too small leads to high variance; too large reintroduces bias. Bickel and Sakov (2008)[10] proposed a data-driven solution by selecting m based on the stability of the bootstrap distribution across multiple values of m, using metrics like the Kolmogorov–Smirnov distance to detect when the distribution "stabilize". In other words, we want to find an optimal m^* such that the limiting distribution of the bootstrap approximates the true generative distribution.

In order to perform the m-out-of-n bootstrap, the covergence rate τ_n^2 of the estimator needs to be known or estimated. If τ_n is assumed to be of the form $\tau_n = n^\epsilon$, we can estimate ϵ by running a linear regression of $log[\hat{Var}(\hat{\boldsymbol{\mu}}^m)]$ on $-2log(m)$ where the variance $var(\hat{\boldsymbol{\mu}}^m)$ can be estimated by sampling with multiple subsampling sizes m.[11] We present the m-out-of-n bootstrap algorithm in Table C6. Based on the findings of Chakraborty et al.,[12] we define $q = 0.95$ and $K = 14$, which implies that the minimum value of m is approximately half the total sample size.

We report the coverage rates and mean width of confidence intervals for Scenario 1(a) (low dimensional covariates with two time-points) with sample size of 200, 500, and 1000, and Scenario 2 (high dimensional covariates with two time-points) with sample size of 500 and 1000 in Table C7. We also evaluate the performance of the naive bootstrap (n-out-of-n bootstrap). Due to computational burden, a total of 200 replicates were conducted for each scenario and each sample size, and within each replicate, the m-out-of-n bootstrap was applied with 200 bootstrap samples. The results demonstrate that the m-out-of-n bootstrap generally achieves higher coverage rates than the naive bootstrap across both scenarios and all sample sizes although

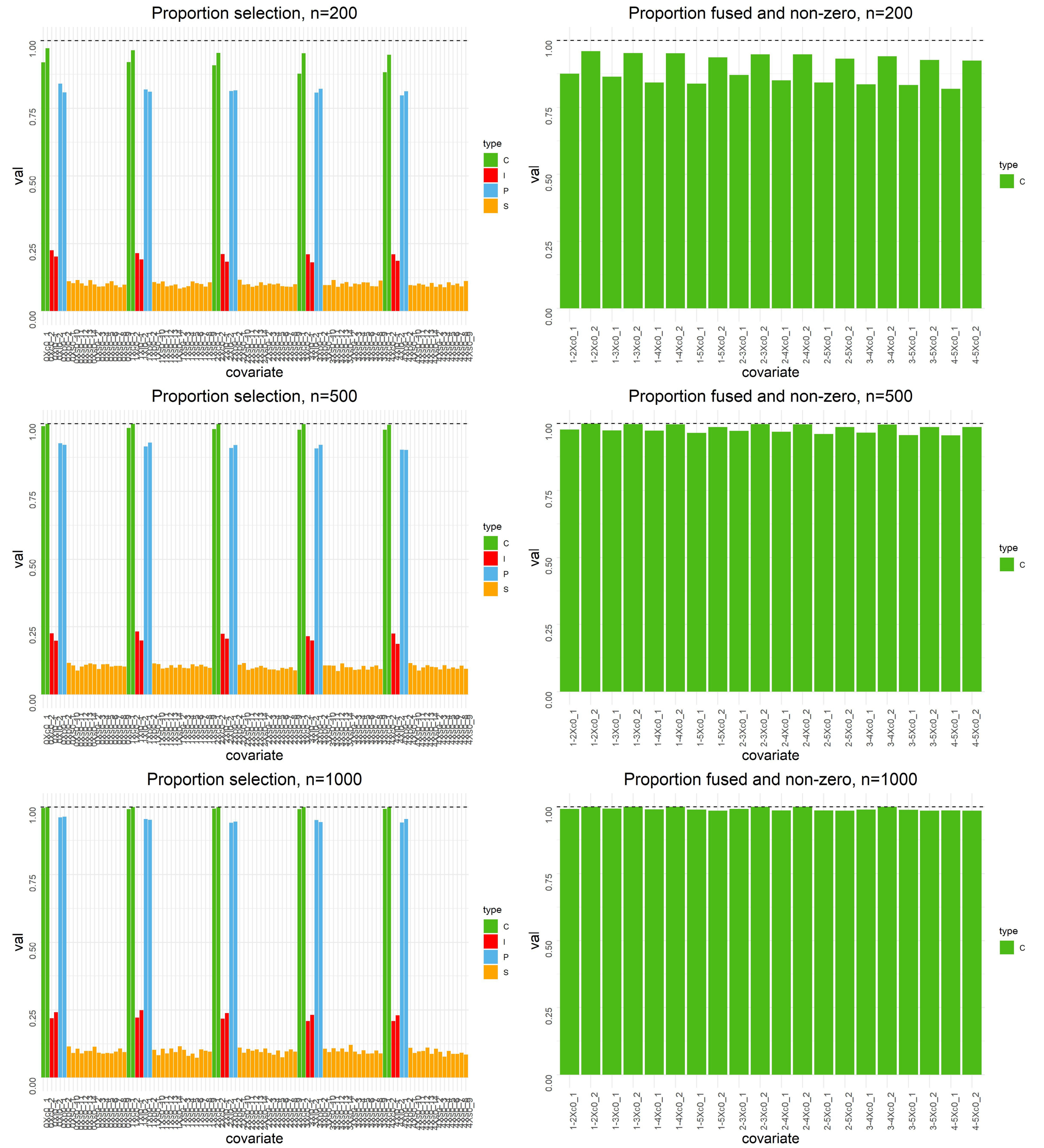

FIGURE C3 Proportion variable selection (left) and fusion of the confounders (right) for n = 200 (top), n = 500 (middle), and n = 1000 (bottom) in Scenario 3. Each bar in the right-hand plots represents the proportion of fusion of each confounder at each pair of time-points. Labels: C are the confounders (should be selected), P are the pure causes of the outcome (should be selected), I are the instruments, and S are noise variables.

confidence intervals via the naive bootstrap have the smaller average width. For example, in Scenario 2 with n=1000, coverage rates under the m/n bootstrap range from 0.83 to 0.84, compared to 0.71 to 0.78 under the naive method.

TABLE C5 $\sqrt{n}$ times absolute value of bias (n times mean squared error) for the estimation of $E(Y^{(1,1)})$ of C-LTMLE, LTMLE with propensity score covariates selected by LOAL, LTMLE with no variable selection, and LTMLE with superlearner (SL).

Method\Scenario	1(a)	1(b)	1(c)	2
$E(Y^{(1,1)})$ true values	1.0	3.5	8.5	1.9
**n=200**				
LTMLE full	0.6(48)	10.2(317)	13.0(1619)	4.0(316)
LTMLE SL	0.6(13)	13.0(308)	15.4(839)	2.2(26)
C-LTMLE	0.3(21)	6.7(268)	10.4(1125)	0.6(18)
LTMLE LOAL	0.4(17)	5.2(215)	8.6(706)	1.9(71)
**n=500**				
LTMLE full	0.8(53)	13.2(617)	20.2(2520)	4.1(287)
LTMLE SL	0.8(14)	22.3(678)	26.0(1422)	2.2(28)
C-LTMLE	0.3(19)	10.8(409)	17.6(1444)	1.2(23)
LTMLE LOAL	0.4(19)	9.2(348)	11.7(1126)	2.0(63)
**n=1000**				
LTMLE full	0.8(65)	17.0(844)	29.6(4516)	3.8(320)
LTMLE SL	0.5(13)	35.8(1478)	43.9(2961)	2.1(27)
C-LTMLE	1.3(21)	14.0(630)	24.7(2022)	1.9(33)
LTMLE LOAL	0.4(22)	12.0(489)	17.4(1956)	2.0(70)

TABLE C6 m-out-of-n bootstrap algorithm

Step	Description
1	Estimate the parameters of the marginal structural model (MSM) using the full sample. Denote this estimate by $\hat{\boldsymbol{\mu}} = (\hat{\mu}_0, \hat{\mu}_1, \hat{\mu}_2)$.
2	For each $j = 0, \dots, K$
2.1	Set the bootstrap sample size as $m_j = \lfloor q^j \cdot n \rfloor$ where $\lfloor x \rfloor$ denotes the largest integer smaller than x and q is a predefined tuning parameter.
2.2	For $b = 1, \dots, B$, draw a sample of size m_j with replacement from the original data of size n.
2.3	Estimate the target parameter $\boldsymbol{\mu}$ from this sample, and denote it $\hat{\boldsymbol{\mu}}[jb] = (\hat{\mu}_0[jb], \hat{\mu}_1[jb], \hat{\mu}_2[jb])$.
2.4	For each parameter $\mu_l \in \boldsymbol{\mu}, l = 0, 1, 2$, estimate the variance of each bootstrap resampled estimate, denoted $var^j(\mu_l) = var(\hat{\mu}_l[jb])$.
3	For each parameter $\mu_l \in \boldsymbol{\mu}, l = 0, 1, 2,$
3.1	For each j, compute the empirical cumulative distribution function of $\sqrt{m_j}(\hat{\mu}_l[jb] - \hat{\mu}_l)$, and denote it by $R_{m_j}(x, \hat{\mu}_l) = \frac{1}{B}\sum_{b=1}^{B} \mathbb{I}\{\sqrt{m_j}(\hat{\mu}_l[jb] - \hat{\mu}_l) \leq x\}$.
3.2	Data-adaptively select an optimal m_j, denoted m^*, which produces the minimum value of the sup-norm of the successive differences between the bootstrap empirical distribution functions, i.e., $m^* = \arg\min_{m_j}\left\{\sup_x \left\|R_{m_j}(x, \hat{\mu}_l) - R_{m_{j+1}}(x, \hat{\mu}_l)\right\|\right\}$ and j^* refers to the corresponding index such that $m^* = m_{j^*}$.
3.3	Run a simple linear regression of $log[var^j(\mu_l)]$ on $-2log(m_j)$ to obtain the coefficient ϵ_l.
3.4	Compute the 95% confidence intervals by calculating $\hat{\mu} \pm 1.96(\frac{m^*}{n})^{\epsilon_l}\sqrt{var^{j^*}(\hat{\mu}_l)}$.

C.6 Evaluating positivity violations

In order to show the extent of practical positivity violations in Scenarios 1 and 2, in Table C8 we provide summaries of the cumulative products of the treatment probabilities used for the estimation of the MSM parameters. These probabilities were estimated first using a logistic regression conditional on all terms (“full model") and then conditional on just those selected by LOAL (“LOAL"), with data corresponding to 200 draws of sample size $n = 500$. Given that the data generating mechanism for treatment is identical in Scenarios (a-c), the corresponding results were the same in the absence of selection. The full model yielded minimum cumulative treatment probabilities of 0.000 in both Scenarios 1 and 2, indicating severe practical positivity violations. In contrast, LOAL mitigated this issue to some extent, with minimum scores ranging from 0.002 to 0.003 across all

TABLE C7 Coverage rates and mean width of 95% confidence intervals for the parameters estimated using LOAL in 200 simulations of Scenario 1(a) (low dimensional with two time points) and of Scenario 2 (high dimensional with two time points)

Secenario	Parameters	Coverage rate in naive bootstrap	Coverage rate in m/n bootstrap	CI width in naive bootstrap	CI width in m/n bootstrap
S1(a)	**n=200**				
	μ_0	0.86	0.88	0.62	0.76
	μ_1	0.85	0.88	0.49	0.60
	μ_2	0.94	0.96	0.49	0.60
	**n=500**				
	μ_0	0.82	0.88	0.42	0.51
	μ_1	0.82	0.89	0.32	0.39
	μ_2	0.90	0.92	0.34	0.41
	**n=1000**				
	μ_0	0.85	0.89	0.31	0.38
	μ_1	0.83	0.90	0.24	0.29
	μ_2	0.87	0.90	0.26	0.31
S2	**n=500**				
	μ_0	0.81	0.88	0.80	0.97
	μ_1	0.81	0.87	0.52	0.64
	μ_2	0.78	0.82	0.61	0.75
	**n=1000**				
	μ_0	0.77	0.84	0.63	0.73
	μ_1	0.78	0.83	0.41	0.47
	μ_2	0.71	0.83	0.48	0.56

scenarios. LOAL yielded slightly lower quantile values (10%–90%), compared to the full model across all scenarios, which indicates that LOAL shifted the bulk of the cumulative probabilities downwards.

TABLE C8 Summary of the cumulative probabilities of treatment using all covariates ("full model") vs selection by LOAL in Scenarios 1 and 2 with 200 draws of $n = 500$

Scenario	Method	Min.	10%	25%	50%	75%	90%	Max.
S1(a)	Full	0.000	0.134	0.262	0.494	0.757	0.897	1
	LOAL	0.003	0.135	0.202	0.403	0.677	0.830	0.998
S1(b)	Full	0.000	0.134	0.262	0.494	0.757	0.897	1
	LOAL	0.003	0.136	0.203	0.402	0.675	0.827	0.997
S1(c)	Full	0.000	0.134	0.262	0.494	0.757	0.897	1
	LOAL	0.002	0.124	0.206	0.406	0.646	0.797	0.998
S2	Full	0.000	0.163	0.339	0.591	0.813	0.928	1
	LOAL	0.003	0.142	0.245	0.417	0.640	0.802	0.999

To investigate the impact of practical positivity violations in the simulation study, we focused on Scenario 1(a) and varied the intercept and the coefficient of I_0 in the probability function of A_0. More specifically, A_0 follows the Bernoulli distribution with probability $\text{logit}(p) = \nu_0 + 1.515C_0 + \nu_I I_0$ where we set the intercept ν_0 to 21 values over the range $[-1.5, 1.5]$ in increments of 0.15, and the coefficient of I_0, ν_I, to 21 values in $[0, 2]$ in increments of 0.1. Therefore, increasing ν_I amplifies the effect of I_0 on the probability of A_0, inducing greater variability across units and pushing propensity scores closer to the boundaries of 0 and 1. Varying the intercept ν_0 shifts the overall probability of A_0.

Figure C4 displays the n times the mean squared error (nMSE) over 200 draws for the estimated parameters μ_0, μ_1, μ_2 using full model and model selected by LOAL across the full range of ν_0 and ν_I, the intercept and coefficient of the instrument I_0 in the treatment generating model respectively, in Scenario 1(a). As the strength of the instrument (ν_I) increased, the performance of full model deteriorated, particularly for μ_0, μ_2 as indicated by rising nMSE. LOAL, in contrast, reduced in nMSE exhibiting robustness in the presence of varying degrees of positivity violations due to instruments. When varying the treatment generating model intercept ν_0, LOAL had consistently better performance than using full model, though its performance was hampered by larger positive values of ν_0.

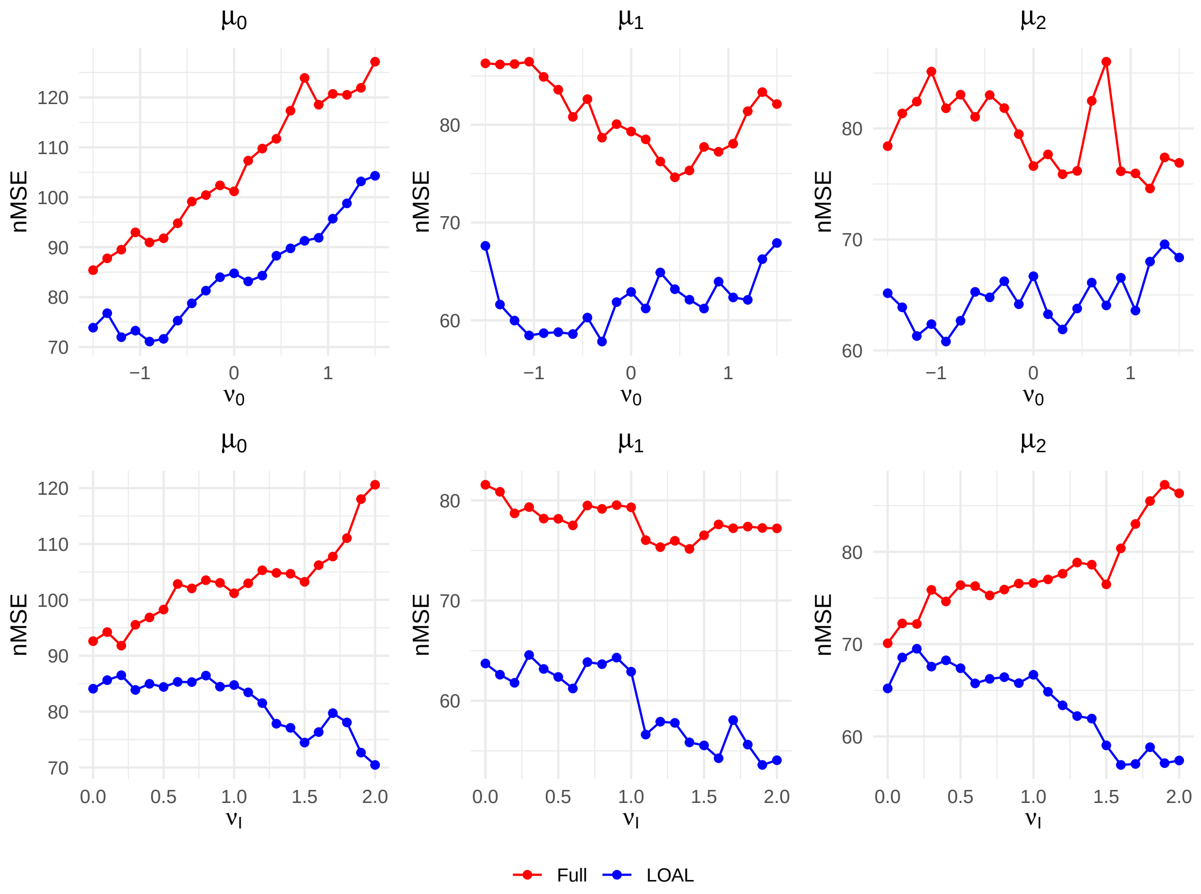

FIGURE C4 n times MSE for parameters over 200 draws when varying treatment data generating model intercept ν_0 (top row) and instrument coefficient ν_I (bottom row) in Scenario 1(a), $n = 500$. Results are shown using full model (red) and model selected by LOAL (blue).

D COMPLETE REPORT ON THE NDIT STUDY ANALYSIS

The Nicotine Dependence in Teens (NDIT) study is a prospective longitudinal study of 1,294 grade 7 students recruited from 10 Montréal-area (Canada) high schools in 1999-2000.[(13)] Self-report questionnaires were administered from grade 7 to 11 at each of the 10 schools every three months for a total of 20 cycles from 1999 to 2005 (i.e., during the five years of high school). Mail or in-person questionnaires were administered in 2007/2008 (cycle 21) when participants were age 20.4 years on average. The data collected include repeated measures of a wide range of socio-demographic, substance use, psychosocial, lifestyle, and physical and mental health variables.

D.1 NDIT data

D.1.1 Exposure

Participants were asked "During the past three months, how often did you drink alcohol (beer, wine, hard liquor)?" We considered a participant exposed to regular alcohol use if the participant answered "once or a couple of times a week" or "usually every day" (alternatives were "never," "a bit to try" or "once or a couple of times a month"). Therefore, "alcohol use" in this paper refers to "at least weekly use". In defining the population of interest, we excluded all participants reporting regular alcohol use at time zero. We denoted the binary exposure over time as A_t for time t.

D.1.2 Censoring

We denote the censoring indicators as C_t for each time t. A participant was censored by time t, denoted $C_t = 1$, when they were lost to follow-up or when they skipped more than one entire year of follow-up; otherwise, $C_t = 0$.

D.1.3 Covariates

Baseline covariates As baseline variables, we included socio-demographic characteristics including sex (with male as the reference sex), mothers' education (no university vs. some university), whether the participant lived in a single-parent home, if the participant spoke French at home, and country of birth (outside Canada vs. Canada), which were assessed in the first data collection cycle. We also included: self-esteem, impulsivity, and novelty-seeking (a genetic tendency to feel intense excitement and actively explore new or potentially rewarding experiences, while also avoiding monotony and possible punishment[(14)]). While these three variables were measured in the 12-th cycle, because they are considered personality traits and unlikely to vary considerably over time, they were included as baseline covariates[(15)]. Self-esteem was measured using Rosenberg's Self-Esteem Scale[(16)]; higher values indicate higher self-esteem.[(17)] Impulsivity was measured with an abbreviated version of the Eysenck Impulsivity Scale,[(18)] which was previously validated among adolescents[(19)]; higher scores indicate greater impulsivity.[(17)] Novelty-seeking was assessed using nine items based on Cloninger's Tridimensional Personality Questionnaire[(20)]; high scores indicate greater novelty-seeking.[(17)]

Time-varying covariates The time-varying covariates $\boldsymbol{L}_t$ were measured for time t and included: Current depressive symptoms; participation in team sports; family-related stress (validated 4-point scale) with higher values indicating more stress; other type of stress (validated 4-point scale); worry about weight; and ever smoked. Unlike the outcome, current depressive symptoms were measured with a validated six-item symptoms scale[(21,22)]; higher scores indicated higher levels of depressive symptoms. Family stress was measured using a validated scale over the past three months; higher values indicate higher levels of stress.[(17)] Other stress referred to the past three months with higher values indicating higher levels of stress.

D.1.4 Outcomes

The outcome Y, depression symptoms, was measured using the Major Depressive Inventory (MDI) in 2007/2008.[(23,24)] Participants asked how much time in the past two weeks they had: 1) felt low in spirit; 2) lost interest in or could no longer enjoy their daily activities; 3) felt a lack of energy and strength; 4) felt less confident; 5) had a bad conscience or feelings of guilt; 6) felt life was not worth living; 7) had difficulty concentrating; 8) felt very restless; 9) felt subdued or slowed down; 10) had trouble sleeping at night or waking up too early; 11) suffered from reduced appetite; and, 12) suffered from increased appetite. A score of four or more for items 1) and 2), and a score of three or more for the other items indicated a diagnostic demarcation for the depression symptom. For items 8) and 9), the highest score was retained for scoring, and similarly for items 11) and 12). Based on a 6-point scale ranging from 0 (*no time*) to 5 (*all the time*) for each item, responses were summed to generate a continuous score from 0 to 50 with higher scores indicating more severe symptoms.[(21,24)] This scale measures depression symptoms over the past two weeks.

D.2 Handling covariate missingness in the analysis

We addressed missing values in covariates that were unrelated to censoring using imputation methods (For cases where missing data were due to censoring, we applied IPTW or LTMLE with censoring weights). To impute missing values in time-dependent covariates, we employed the Last Observation Carried Forward method which was applied for no more than one full academic year of follow-up after the last measured value. For handling missing data in baseline and the remaining time-varying covariates, we utilized multiple imputations by chained equations (MICE), mice `R` package,[(25)] maintaining time-ordering of the follow-up variables throughout this process. Our imputation process involved a single database, and subsequent analyses were conducted to derive the estimates.

D.3 Target trial

We aim to study the effect of time of initiation of drinking during early high school on depression in young adulthood. To do so, we analyzed data from the NDIT study collected over a span of the first five cycles from 1999 to 2000 and the 21st cycle in 2007/2008. We define the target trial with corresponding intention-to-treat parameters of interest. This hypothetical trial recruits participants who have not yet initiated regular drinking at the beginning of grade 7, e.g. $A_0 = 0$ for all participants. The target trial randomizes drinking initiation to one of the follow-up time points and conducts an analysis of the correlation between time of initiation and depression symptoms in young adulthood Y.

D.4 Parameter of interest

To perform the intention-to-treat analysis with our observational data, we defined the exposure variable such that once an individual was exposed to regular alcohol use, they were considered exposed for the duration of the study unless they were lost to follow-up. Specifically, if an individual had a value of 1 for A_t at any $t = 1, \cdots, 5$, we coded the variable to set all subsequent time points, A_{t+k}, to 1 for $k = 1, \cdots, 6 - t$. Define $\mathcal{D}$ as the regimen space for the intention-to-treat analysis, then $\mathcal{D}$ represents 6 treatment patterns where the initiation time varies between 1 and 5, and no initiation for all time points, i.e.

$$\mathcal{D} = \begin{Bmatrix} (1, & 1, & 1, & 1, & 1) \\ (0, & 1, & 1, & 1, & 1) \\ (0, & 0, & 1, & 1, & 1) \\ (0, & 0, & 0, & 1, & 1) \\ (0, & 0, & 0, & 0, & 1) \\ (0, & 0, & 0, & 0, & 0) \end{Bmatrix}.$$

Then the parameters of interest can be defined through the working MSM,

$$\mathbb{E}[Y^{\bar{a}}|\text{Sex}] = \mu_0 + \mu_1\text{Sex} + \mu_2\text{cum}(\bar{\boldsymbol{a}}) + \mu_3\{\text{Sex} \times \text{cum}(\bar{\boldsymbol{a}})\}$$

where $\mathbb{E}[Y^{\bar{a}}|\text{sex}]$ represents the mean counterfactual outcome under some intervention pattern $\bar{\boldsymbol{a}}$ in a sex subgroup such that sex=1 denotes female, and $\text{cum}(\bar{\boldsymbol{a}})$ counts the cumulative exposures in the pattern. The true parameter values $\boldsymbol{\mu}$ minimize the expectation of a squared error loss function, summing over all patterns in the intention-to-treat space $\mathcal{D}$, corresponding to the parameters estimated in the hypothetical target trials.

D.5 Model specification

D.5.1 Data structure

Given the above, the following represents the observed data structure:

$$O = \{\boldsymbol{L}_1, A_1, \boldsymbol{L}_2, C_2, A_2, \cdots, A_5, \boldsymbol{L}_6, C_6, Y\}.$$

Note that $\boldsymbol{L}_1$ contains the baseline covariates and the time-varying covariates at the first time and there is no censoring prior to the first exposure time.

D.5.2 Outcome models

We use the notation $\bar{\boldsymbol{L}}_t$ to denote the history of baseline and time-dependent covariates up to time t and likewise $\bar{\boldsymbol{A}}_t$ represents the history of the exposure $A_1, \cdots, A_t$. We rescaled the bounded continuous outcome Y to be contained in $(0, 1)$. Denote $T = 6$ as the total number of time points. Starting with $q_{T+1}(\bar{\boldsymbol{a}}_{T+1}, \bar{\boldsymbol{L}}_{T+1}) = Y$, we recursively define

$$q_t(\bar{\boldsymbol{a}}_t, \bar{\boldsymbol{L}}_t) = \mathbb{E}\{q_{t+1}(\bar{\boldsymbol{a}}_{t+1}, \bar{\boldsymbol{L}}_{t+1})|\bar{\boldsymbol{L}}_t, C_t = 0, \bar{\boldsymbol{A}}_t = \bar{\boldsymbol{a}}_t\}, \quad t = T, \cdots, 1.$$

For the NDIT data setting, the history of the exposures up to time T is equal to the history of the exposures to time $T - 1$. In order to obtain preliminary estimates $q_{t,n}(\bar{\boldsymbol{a}}_t, \bar{\boldsymbol{L}}_t)$ of $q_t(\bar{\boldsymbol{a}}_t, \bar{\boldsymbol{L}}_t)$, we modeled the outcome for $t = T$ or most recent estimate of $q_{t+1}(\bar{\boldsymbol{a}}_t, \bar{\boldsymbol{L}}_t)$, conditional on main terms of the baseline and time varying covariates, exposure terms (current and lagged) and the first-order interactions of sex and exposure terms for uncensored participants. Then we generated predictions from this model for each pattern of interest $\bar{\boldsymbol{a}}$. We fit logistic regressions stratified on time t, corresponding to:

For $t = T$,

$$Y \sim \sum_{k=1}^{t} \boldsymbol{L}_k + \sum_{k=1}^{t-1} A_k + \sum_{k=1}^{t-1} \{\text{Sex} \times A_k\}$$

For $t = T - 1, \cdots, 1$,

$$q_{t+1,n}(\bar{\boldsymbol{a}}) \sim \sum_{k=1}^{t} \boldsymbol{L}_k + \sum_{k=1}^{t} A_k + \sum_{k=1}^{t} \{\text{Sex} \times A_k\}.$$

Taking as outcome a vector composed of stacked components $q_{t,n}(\bar{\boldsymbol{a}}_t, \bar{\boldsymbol{L}}_t)$ for each pattern $\bar{\boldsymbol{a}}$, we then run regressions according to the following working regression models:

$$\begin{aligned}
&\hat{E}\{q_{1,n} \mid \bar{\boldsymbol{L}}_1, a_1\} = \beta_{1,0} + \boldsymbol{\beta}_{1,1}\boldsymbol{L}_1 + \beta_{1,2}a_1,\\
&\hat{E}\{q_{2,n} \mid \bar{\boldsymbol{L}}_2, C_2 = 0, \bar{\boldsymbol{a}}_2\} = \beta_{2,0} + \boldsymbol{\beta}_{2,1}\boldsymbol{L}_1 + \boldsymbol{\beta}_{2,2}\boldsymbol{L}_2 + \beta_{2,3}a_1 + \beta_{2,4}a_2,\\
&\vdots\\
&\hat{E}\{q_{T,n} \mid \bar{\boldsymbol{L}}_T, C_T = 0, \bar{\boldsymbol{a}}_T\} = \beta_{T,0} + \sum_{k=1}^{T} \boldsymbol{\beta}_{T,k}\boldsymbol{L}_k + \sum_{k=1}^{T} \beta_{T,T+k}a_k
\end{aligned} \tag{D2}$$

with true parameter values $\boldsymbol{\beta} = \{\beta_{\tau,t}; \tau = (1, \cdots, T),\ t = (0, \cdots, 2\tau)\}$ minimizing the risk under a squared-error loss function.

D.5.3 Pooled treatment model and pooled censoring model

As discussed in the manuscript, we define a "full" model for the probability of treatment at all time-points that adjusts for the full covariate history. The treatment model was fit using those who had not yet initiated and were uncensored at each time point. A pooled logistic regression for the conditional probability of treatment, $P(A_t = 1 \mid A_{t-1} = 0, \bar{\boldsymbol{L}}_t, C_t = 0)$, was specified as follows. We define $m_t(\bar{\boldsymbol{L}}_t; \boldsymbol{\alpha})$ as the corresponding model for the probability of treatment at times $t = 1, \cdots, T$.

$$\begin{aligned}
&\text{logit}\left\{m_t(\bar{\boldsymbol{L}}_t; \boldsymbol{\alpha})\right\}\\
&= \mathbb{I}(t = 1)\left(\alpha_{1,0} + \boldsymbol{\alpha}_{1,1}\boldsymbol{L}_1\right) +\\
&\quad \mathbb{I}(t = 2)\left(\alpha_{2,0} + \boldsymbol{\alpha}_{2,1}\boldsymbol{L}_1 + \boldsymbol{\alpha}_{2,2}\boldsymbol{L}_2\right) +\\
&\quad \vdots\\
&\quad \mathbb{I}(t = T - 1)\left(\alpha_{T-1,0} + \sum_{k=1}^{T-1} \boldsymbol{\alpha}_{T-1,k}\boldsymbol{L}_k\right).
\end{aligned}$$

In the above, $\boldsymbol{\alpha} = \{\alpha_{\tau,t}; \tau = (1, \cdots, T - 1),\ t = (0, \cdots, \tau)\}$ are the coefficients of the covariates in the pooled propensity score model. Notably, the exposure does not appear in this model since it is fit on the subset of participants who had not yet initiated drinking (and so all past exposure is null).

In addition, the pooled censoring model adjusted for the history of covariates and treatments in order to estimate $h_t(\bar{\boldsymbol{L}}_t, \bar{A}_{t-1}; \boldsymbol{\theta}) = P(C_t = 0 \mid \bar{\boldsymbol{L}}_t, \bar{A}_{t-1}, C_{t-1} = 0); t = 2, \cdots, T$. The model was specified as

$$\begin{aligned}
&\text{logit}\left\{1 - h_t(\bar{\boldsymbol{L}}_t, \bar{A}_{t-1}, ; \boldsymbol{\theta})\right\}\\
&= \mathbb{I}(t = 2)\left(\theta_{2,0} + \boldsymbol{\theta}_{2,1}\boldsymbol{L}_1 + \boldsymbol{\theta}_{2,2}\boldsymbol{L}_2 + \theta_{2,3}A_1\right) +\\
&\quad \vdots\\
&\quad \mathbb{I}(t = T)\left(\theta_{T,0} + \sum_{k=1}^{T} \boldsymbol{\theta}_{T,k}\boldsymbol{L}_k + \sum_{k=1}^{T-1} \theta_{T,T+k}A_k\right).
\end{aligned}$$

In the above, $\boldsymbol{\theta} = \{\theta_{\tau,t}; \tau = (2, \cdots, T),\ t = (0, \cdots, 2\tau - 1)\}$ are the coefficients of the covariates in the pooled censoring model.

D.6 Cumulative weights for treatment and censoring

To define the weights used in estimation and the balance criteria, we must extend our definition of the model for the probability of exposure to be deterministic when exposure was already initiated in the past. Thus, we define

$$m_t^*(\bar{\boldsymbol{L}}_t, a_{t-1}; \boldsymbol{\alpha}) = \begin{cases} m_t(\bar{\boldsymbol{L}}_t; \boldsymbol{\alpha}) & \textit{for } a_{t-1} = 0 \\ 1 & \textit{for } a_{t-1} = 1. \end{cases}$$

The cumulative weights for treatment at times $t = (1, \cdots, T-1)$ are

$$\begin{aligned} w_t^a(a_t, \bar{\boldsymbol{L}}_t; \boldsymbol{\alpha}) &= \frac{\mathbb{I}(A_t = a_t)}{a_t m_t(\bar{\boldsymbol{L}}_t; \boldsymbol{\alpha}) + (1-a_t)[1 - m_t(\bar{\boldsymbol{L}}_t; \boldsymbol{\alpha})]} \quad \text{for } t = 1, \text{and} \\ w_t^a(a_t, \bar{\boldsymbol{a}}_{t-1}, \bar{\boldsymbol{L}}_t; \boldsymbol{\alpha}) &= \frac{\mathbb{I}(A_t = a_t, \bar{\boldsymbol{A}}_{t-1} = \bar{\boldsymbol{a}}_{t-1})}{\prod_{k=1}^t a_k m_k^*(a_{k-1}, \bar{\boldsymbol{L}}_k; \boldsymbol{\alpha}) + (1-a_k)[1 - m_k^*(a_{k-1}, \bar{\boldsymbol{L}}_k; \boldsymbol{\alpha})]} \quad \text{for } t = 2, \cdots, T-1. \end{aligned} \tag{D3}$$

The cumulative weight for censoring at time $t = (2, \cdots, T)$ is

$$w_t^c(\bar{\boldsymbol{L}}_t, \bar{\boldsymbol{a}}_{t-1}; \boldsymbol{\theta}) = \prod_{k=2}^{t} \frac{\mathbb{I}(C_k = 0)}{h_k(\bar{\boldsymbol{L}}_k, \bar{\boldsymbol{a}}_{k-1}; \boldsymbol{\theta})}. \tag{D4}$$

Combinations of these weights are used in the balance criteria.

The cumulative weights used in IPTW are written as: $w_t^{iptw} = 1/P(A_t = a_t \mid \boldsymbol{L}_t = \boldsymbol{l}_t)$ for time $t = 1$ and $w_t^{iptw} = 1/P(A_1 = a_1 \mid \boldsymbol{L}_1 = \boldsymbol{l}_1) \prod_{k=2}^t w_k$ for time $t = (2, \cdots, T)$ where

$$w_k = \begin{cases} \dfrac{\mathbb{I}(C_k = 0)}{P(A_k = a_k \mid a_{k-1} = 0, \bar{\boldsymbol{L}}_k = \bar{\boldsymbol{l}}_k, C_k = 0) P(C_k = 0 \mid \bar{\boldsymbol{L}}_k = \bar{\boldsymbol{l}}_k, C_{k-1} = 0, a_{k-1} = 0)} & \textit{for } a_{k-1} = 0 \\ \dfrac{\mathbb{I}(C_k = 0)}{P(C_k = 0 \mid \bar{\boldsymbol{L}}_k = \bar{\boldsymbol{l}}_k, C_{k-1} = 0, \bar{\boldsymbol{A}}_{k-1} = \bar{\boldsymbol{a}}_{k-1})} & \textit{for } a_{k-1} = 1. \end{cases}$$

D.7 Longitudinal Outcome Adaptive Lasso

We implemented LOAL to select variables for the treatment models and censoring models separately at each time that have corresponding non-zero coefficients $\boldsymbol{\beta}$ in the q_t model fits.

D.7.1 LOAL for treatment

Given a regularization parameter $\lambda_n^a \geq 0$, the pooled LOAL estimator for $\boldsymbol{\alpha}^\dagger$ is given as,

$$\begin{aligned} \hat{\alpha}(\lambda_n^a) = \arg\min_{\alpha} \sum_{\tau=1}^{T} \sum_{i=1}^{n} \big[& a_{\tau,i} \log\{m_\tau(\bar{\boldsymbol{l}}_{\tau,i}, \bar{\boldsymbol{a}}_{\tau-1,i}; \boldsymbol{\alpha})\} \\ & + (1 - a_{\tau,i}) \log\{1 - m_\tau(\bar{\boldsymbol{l}}_{\tau,i}, \bar{\boldsymbol{a}}_{\tau-1,i}; \boldsymbol{\alpha})\}\big] + \lambda_n^a \sum_{j \in \mathcal{J}^a} \hat{\omega}_j |\alpha_j|, \end{aligned}$$

where $\hat{\omega}_j = |\hat{\beta}_j|^{-\gamma}$ for $j \in \mathcal{J}^a$ and $\gamma = 2.5$. Here $\mathcal{J}^a$ represents the indices of the coefficients $\boldsymbol{\alpha}$ being shrunk, i.e.,

$$\begin{aligned} \mathcal{J}^a = \{ & (1, 1, \mathcal{J}_{1,1}^a), \\ & (2, 1, \mathcal{J}_{2,1}), (2, 2, \mathcal{J}_{2,2}^a), \\ & \vdots \\ & (T-1, 1, \mathcal{J}_{T-1,1}^a), (T-1, 2, \mathcal{J}_{T-1,2}^a), \cdots, (T-1, T-1, \mathcal{J}_{T-1,T-1}^a)\} \end{aligned}$$

where $\mathcal{J}_{\tau,t}^a$, for $\tau, t = (1, \cdots, T-1), t \leq \tau$, represents the indices of set of coefficients at each time. Specifically, $\mathcal{J}_{\tau,t}^a$ indexes the specific covariates in $\boldsymbol{L}_t$ being shrunk within propensity score model $\boldsymbol{A}_\tau$.

This regularized regression for the treatment can be implemented by a transformation of the pooled data, setting

$$\begin{aligned}
&V_{1,0} = \mathbb{I}(t = 1), \boldsymbol{V}_{1,1} = \mathbb{I}(t = 1)\boldsymbol{L}_1,\\
&V_{2,0} = \mathbb{I}(t = 2), \boldsymbol{V}_{2,1} = \mathbb{I}(t = 2)\boldsymbol{L}_1, \boldsymbol{V}_{2,2} = \mathbb{I}(t = 2)\boldsymbol{L}_2,\\
&\vdots\\
&V_{T-1,0} = \mathbb{I}(t = T-1), \boldsymbol{V}_{T-1,1} = \mathbb{I}(t = T-1)\boldsymbol{L}_1, \cdots, \boldsymbol{V}_{T-1,T-1} = \mathbb{I}(t = T-1)\boldsymbol{L}_{T-1}
\end{aligned}$$

with respectively corresponding coefficients in $\boldsymbol{\alpha}$. Then, the adaptive LASSO is run with pooled outcome A_τ on these covariates $V_{1,0}, ..., \boldsymbol{V}_{T-1,T-1}$, without an intercept term, using weights $\hat{\omega}_j = |\hat{\beta}_j|^{-\gamma}; j \in \mathcal{J}^a$.

D.7.2 LOAL for censoring

For the censoring, given the same $\hat{\omega}_j = |\hat{\beta}_j|^{-\gamma}$ for $j \in \mathcal{J}^c$ and $\gamma = 2.5$, the pooled LOAL estimator $\boldsymbol{\theta}_0^\dagger$ on $\lambda_n^c \geq 0$ is,

$$\begin{aligned}
\hat{\boldsymbol{\theta}}(\lambda_n^c) = \arg\min_{\theta} \sum_{\tau=2}^{T} \sum_{i=1}^{n} \big[&(1 - c_{\tau,i}) \log\{h_\tau(\bar{\boldsymbol{l}}_{\tau,i}, \bar{\boldsymbol{a}}_{\tau-1,i}; \boldsymbol{\theta})\}\\
&+ c_{\tau,i} \log\{1 - h_\tau(\bar{\boldsymbol{l}}_{\tau,i}, \bar{\boldsymbol{a}}_{\tau-1,i}; \boldsymbol{\theta})\}\big] + \lambda_n^c \sum_{j \in \mathcal{J}^c} \hat{\omega}_j |\theta_j|,
\end{aligned}$$

where $\mathcal{J}^c$ represents the indices of the coefficients $\boldsymbol{\theta}$ being shrunk. $\mathcal{J}^c_{\tau,t}$ for $\tau = (2, \cdots, T), t = (1, \cdots, T), t \leq \tau$ indexes the specific covariates in $\boldsymbol{L}_t$ within the censoring models C_τ. Note that the intercepts and the coefficients corresponding to treatments are not shrunk,

$$\begin{aligned}
\mathcal{J}^c = \{&(2, 1, \mathcal{J}^c_{2,1}), (2, 2, \mathcal{J}^c_{2,2}),\\
&(3, 1, \mathcal{J}^c_{3,1}), (3, 2, \mathcal{J}^c_{3,2}), (3, 3, \mathcal{J}^c_{3,3}),\\
&\vdots\\
&(T, 1, \mathcal{J}^c_{T,1}), (T, 2, \mathcal{J}^c_{T,2}), \cdots, (T, T, \mathcal{J}^c_{T,T})\}.
\end{aligned}$$

Likewise, this regularization for the censoring can be applied through a transformation of the pooled data, but encompassing not only the variables of time and covariates but also treatment related variables,

$$\begin{aligned}
&U_{2,0} = \mathbb{I}(t = 2), U_{2,1} = \mathbb{I}(t = 2)\boldsymbol{L}_1, U_{2,2} = \mathbb{I}(t = 2)\boldsymbol{L}_2, U_{2,3} = \mathbb{I}(t = 2)A_1,\\
&\vdots\\
&U_{T,0} = \mathbb{I}(t = T), U_{T,1} = \mathbb{I}(t = T)\boldsymbol{L}_1, \cdots, U_{T,T} = \mathbb{I}(t = T)\boldsymbol{L}_T,\\
&\quad U_{T,T+1} = \mathbb{I}(t = T)A_1, \cdots, U_{T,2T-1} = \mathbb{I}(t = T)A_{T-1}
\end{aligned}$$

with respectively corresponding coefficients in $\boldsymbol{\theta}$. Then, the adaptive LASSO is run with pooled outcome C_t on these covariates $U_{2,0}, ..., U_{T,2T-1}$ without an intercept term.

D.8 Selection of λ_n^a and λ_n^c

The tuning parameter λ_n^a and λ_n^c were selected jointly by minimizing the sum of the balance metrics for treatment and censoring. The balance metric for treatment, $\mathcal{M}$, is a summary of weighted absolute mean differences (wAMDs) of the covariates between the exposure groups.[26] Similarly, the balance metric for censoring, $\mathcal{N}$, is based on the wAMDs of the covariates between uncensored and censored groups.

The weights involved are cumulative inverse probability weights for current treatment or censoring. For balance across treatment groups at a given time, we only consider histories with no past exposure, since the only comparison to make is in people who initiated or did not initiate exposure. Let $\hat{\boldsymbol{\alpha}}^{refit}(\lambda_n^a)$ represent the estimates from a logistic regression of the treatment on the covariates selected by LOAL under tuning parameter λ_n^a where the value is set to be zero if the corresponding coefficient was not selected. Similarly, $\hat{\boldsymbol{\theta}}^{refit}(\lambda_n^c)$ represent the estimates from a logistic regression of the censoring on the covariates selected by LOAL under tuning parameter λ_n^c. Based on equations (D3) and (D4), we define the weight for subject i at time t for the

current treatment as

$$w^a_{t,i} = w^a_t\{a_{t,i}, \bar{\boldsymbol{a}}_{t-1,i} = 0, \bar{\boldsymbol{l}}_{t,i}; \hat{\boldsymbol{\alpha}}^{refit}(\lambda^a_n)\} w^c_t\{\bar{\boldsymbol{a}}_{t-1,i}, \bar{\boldsymbol{l}}_{t,i}; \hat{\boldsymbol{\theta}}^{refit}(\lambda^c_n)\}$$

where $\hat{\boldsymbol{\alpha}}(\lambda^a_n)$ and $\hat{\boldsymbol{\theta}}(\lambda^c_n)$ are parameter estimates under the treatment and censoring model after variable selection by LOAL with the tuning parameters $(\lambda^a_n, \lambda^c_n)$, respectively. Also,

$$w^c_{t,i} = w^c_t\{\bar{\boldsymbol{a}}_{t-1,i}, \bar{\boldsymbol{l}}_{t,i}; \hat{\boldsymbol{\theta}}^{refit}(\lambda^c_n)\} w^a_{t-1}\{a_{t-1,i}, \bar{\boldsymbol{l}}_{t-1,i}; \hat{\boldsymbol{\alpha}}^{refit}(\lambda^a_n)\}$$

is the weight for current censoring for subject i at time t estimated under the proposed LOAL approach of censoring and treatment. Let $L_{t,k}$ denote the kth component in $\boldsymbol{L}_t$ for $k = (1, \cdots, p_k)$ where p_k represents the number of components of $\boldsymbol{L}_t$. Then $\beta_{\tau,t,k}$ represents the coefficients in the structural equations (equations D2) for $\tau = (1, \cdots, T-1)$ and $\tau = (2, \cdots, T)$ referring to the treatment model and censoring model, respectively. Then the weighted absolute mean difference of the treatment and of the censoring can be evaluated based on the variable considered at time t respectively weighted by the corresponding structural models coefficient (equations D2) divided by its standard error.

$$\text{wAMD}^a_{\tau,t,k} = \frac{|\hat{\beta}_{\tau,t,k}|}{\sigma_{\hat{\beta}_{\tau,t,k}}} \left| \frac{\sum_{i=1}^n a_{\tau,i} l_{t,k,i} w^a_{\tau,i} \mathbb{I}(a_{\tau-1,i} = 0, c_{\tau,i} = 0)}{\sum_{i=1}^n a_{\tau,i} w^a_{\tau,i} \mathbb{I}(a_{\tau-1,i} = 0, c_{\tau,i} = 0)} - \frac{\sum_{i=1}^n (1 - a_{\tau,i}) l_{t,k,i} w^a_{\tau,i} \mathbb{I}(a_{\tau-1,i} = 0, c_{\tau,i} = 0)}{\sum_{i=1}^n (1 - a_{\tau,i}) w^a_{\tau,i} \mathbb{I}(a_{\tau-1,i} = 0, c_{\tau,i} = 0)} \right|$$

for $\tau = (1, \cdots, T-1), t = (1, \cdots, T-1)$ and $t \le \tau$.

$$\text{wAMD}^c_{\tau,t,k} = \frac{|\hat{\beta}_{\tau,t,k}|}{\sigma_{\hat{\beta}_{\tau,t,k}}} \left| \frac{\sum_{i=1}^n \mathbb{I}(c_{\tau,i} = 0) l_{t,k,i} w^c_{\tau,i}}{\sum_{i=1}^n \mathbb{I}(c_{\tau,i} = 0) w^c_{\tau,i}} - \frac{\sum_{i=1}^n \{\mathbb{I}(c_{\tau,i} = 1) l_{t,k,i} w^c_{\tau,i}\}}{\sum_{i=1}^n \{\mathbb{I}(c_{\tau,i} = 1) w^c_{\tau,i}\}} \right|$$

for $\tau = (2, \cdots, T), t = (1, \cdots, T)$ and $t \le \tau$

We selected the two tuning parameters by minimizing the sum of the balance criterion for the treatment and the balance criterion for the censoring, i.e. the selected $(\lambda^a_n, \lambda^c_n) = \arg\min_{\lambda^a_n, \lambda^c_n}(\mathcal{M} + \mathcal{N})$ where

$$\begin{aligned}
\mathcal{M} &= \sum_{k=1}^{p_1} \text{wAMD}^a_{1,1,k} + \sum_{t=1}^{2}\sum_{k=1}^{p_k} \text{wAMD}^a_{2,t,k} + \cdots + \sum_{t=1}^{T-1}\sum_{k=1}^{p_k} \text{wAMD}^a_{T-1,t,k} \\
&= \sum_{\tau=1}^{T-1}\sum_{t=1}^{\tau}\sum_{k=1}^{p_k} \text{wAMD}^a_{\tau,t,k} \\
\mathcal{N} &= \sum_{t=1}^{2}\sum_{k=1}^{p_k} \text{wAMD}^c_{2,t,k} + \sum_{t=1}^{3}\sum_{k=1}^{p_k} \text{wAMD}^c_{3,t,k} + \cdots + \sum_{t=1}^{T}\sum_{k=1}^{p_k} \text{wAMD}^c_{T,t,k} \\
&= \sum_{\tau=2}^{T}\sum_{t=1}^{\tau}\sum_{k=1}^{p_k} \text{wAMD}^c_{\tau,t,k}
\end{aligned}$$

D.9 Selective fusion

To perform the selective fusion, we initially establish a penalty graph, wherein vertices represent coefficients within the pooled model eligible for fusion. This graph may be structured with cliques connecting elements that share common variable names across different time points. For example, it links the remaining baseline covariates in different treatment model times. As for time-varying covariates, we created cliques to allow coefficient fusion of the most recent variables of common types across various time points; this allows for the possibility of common effects of historical covariates with common lag time on current exposure initiation. Specifically, the penalty graph connected the same baseline variables across time points, and the same time-varying variables with the same lag across time points (e.g., the $\boldsymbol{L}_{t-1}$ variables are connected when modeling treatment and censoring across times t).

For example, consider the propensity score model for A_4 and a particular time varying covariate $L_{t,3}$. Suppose that both $L_{3,3}$ (the most recent) and $L_{4,3}$ (the current) were selected into this model. In the model for A_5, suppose that $L_{4,3}$ and $L_{5,3}$ were selected. In the model for A_6, suppose that $L_{4,3}$ and $L_{6,3}$ were selected. The cliques connect the coefficients of the three current

variables, $L_{4,3}, L_{5,3}$, and $L_{6,3}$ across the models for A_4, A_5 and A_6, respectively, and also connect the two most recent variables, $L_{3,3}$ and $L_{4,3}$ in the models for A_4 and A_5, respectively.

Denote $\mathcal{G}$ as the set of all pairwise connected indices of the coefficients in accordance with the fusion graph definition. The fused LASSO penalizes the absolute differences between the coefficients of connected variables.

Define $\boldsymbol{\alpha}^*$ as the parameter vector of the same length as $\boldsymbol{\alpha}$ that is set to zero at the indices of the zero-elements of $\hat{\boldsymbol{\alpha}}^{refit}(\lambda_n^a)$.Then the generalized Adaptive Fused LASSO for treatments is

$$\arg\min_{\boldsymbol{\alpha}^*} \sum_{\tau=1}^{T-1}\sum_{i=1}^{n}\left[a_{\tau,i}\log\{m_\tau(\bar{\boldsymbol{l}}_{\tau,i},\bar{\boldsymbol{a}}_{\tau-1,i};\boldsymbol{\alpha}^*)\} + (1-a_{\tau,i})\log\{1-m_\tau(\bar{\boldsymbol{l}}_{\tau,i},\bar{\boldsymbol{a}}_{\tau-1,i};\boldsymbol{\alpha}^*)\}\right]$$
$$+\lambda_{1,n}^a \sum_{(\mathcal{J}^a_{\tau,t},\mathcal{J}^a_{\tau',t'})\in\mathcal{G}^a} \frac{|\boldsymbol{\alpha}^*_{\mathcal{J}^a_{\tau,t}} - \boldsymbol{\alpha}^*_{\mathcal{J}^a_{\tau',t'}}|}{|\hat{\boldsymbol{\alpha}}^{refit}_{\mathcal{J}^a_{\tau,t}}(\lambda_n^a) - \hat{\boldsymbol{\alpha}}^{refit}_{\mathcal{J}^a_{\tau',t'}}(\lambda_n^a)|^{\mathcal{T}}}.$$

where $(\mathcal{J}^a_{\tau,t},\mathcal{J}^a_{\tau',t'})$ is a pair of indices in the graph for treatment $\mathcal{G}^a$. Note that τ,τ',t,t' all in $(1,\cdots,T-1)$, and $t\leq\tau, t'\leq\tau', \tau\neq\tau', t\neq t'$, and $\tau-\tau'=t-t'$ based on the penalty graph we defined.

The penalty graph for censoring $\mathcal{G}^c$ was created in the same way as the graph for treatment model. Note that all coefficients corresponding to the treatment terms were not allowed to fuse. Denote $\boldsymbol{\theta}^*$ as the vector of the same length as $\boldsymbol{\theta}$ that is set to zero at the indices of the zero-elements of $\hat{\boldsymbol{\theta}}(\lambda_n^c)$. The generalized Adaptive Fused LASSO for the censoring is

$$\arg\min_{\boldsymbol{\theta}^*} \sum_{\tau=2}^{T}\sum_{i=1}^{n}\left[(1-c_{\tau,i})\log\{h_\tau(\bar{\boldsymbol{l}}_{\tau,i},\bar{\boldsymbol{a}}_{\tau-1,i};\boldsymbol{\theta}^*)\} + c_{\tau,i}\log\{1-h_\tau(\bar{\boldsymbol{l}}_{\tau,i},\bar{\boldsymbol{a}}_{\tau-1,i};\boldsymbol{\theta}^*)\}\right]$$
$$+\lambda_{1,n}^c \sum_{(\mathcal{J}^c_{\tau,t},\mathcal{J}^c_{\tau',t'})\in\mathcal{G}^c} \frac{|\boldsymbol{\theta}^*_{\mathcal{J}^c_{\tau,t}} - \boldsymbol{\theta}^*_{\mathcal{J}^c_{\tau',t'}}|}{|\hat{\boldsymbol{\theta}}^{refit}_{\mathcal{J}^c_{\tau,t}}(\lambda_n^c) - \hat{\boldsymbol{\theta}}^{refit}_{\mathcal{J}^c_{\tau',t'}}(\lambda_n^c)|^{\mathcal{T}}},$$

where $(\mathcal{J}^c_{\tau,t},\mathcal{J}^c_{\tau',t'})$ is a pair of connected indices in the graph for censoring $\mathcal{G}^c$ and τ,τ',t,t' all in $(2,\cdots,T)$, and $t\leq\tau, t'\leq\tau', \tau\neq\tau', t\neq t'$, and $\tau-\tau'=t-t'$. We utilized the archived `FusedLasso` package to implement the fusion step. The selection of optimal $\lambda_{1,n}^a$ and $\lambda_{1,n}^c$ values for the treatment and censoring models was determined based on the summation of the Bayesian Information Criterion (BIC) of the treatment and censoring models.

D.10 NDIT results

The intention-to-treat analysis of the NDIT data included eight baseline covariates and six time-varying covariates. The pooled treatment model included 130 variables, while the pooled censoring model conditioned on 175 variables. To regularize our models, we set the tuning parameter γ to 2.5 and set 20 possible values for the tuning parameters λ^a and λ^c (refer to Table D2). The selected tuning parameters, $[\lambda_n^a, \lambda_n^c]$, were found to be $[3.728, 67.392]$. These values corresponded to a strong penalty for the treatment model and a relatively light penalty for the censoring model. For the fusion step, we considered 20 possible values for $\lambda_{1,n}^a$ within the range $[e^{-10}, e^{-1}]$ and for $\lambda_{1,n}^c$ within the range $[e^{-5}, 1]$. Ultimately, the minimum summation of BICs corresponding to the treatment and censoring was achieved with $\lambda_{1,n}^a$ in $[0.002, 0.368]$ and $\lambda_{1,n}^c$ in $[0.206, 1]$.

The initial treatment model, with 135 parameters (including five intercepts), was reduced to 37 parameters. Consequently, the fusion step further reduced the number of parameters to 23 (see Table in the main manuscript). The variables sex, country of birth, current depressive symptoms, and worry about weight were selected for inclusion in each time period, and their corresponding coefficients were then fused. The variables mother education, ever smoked, family stress, other stress, and team sports were selected into the models for some time-points but were not fused. For the censoring models, initially, there were 180 parameters (including five intercepts and 15 past treatments), which were later reduced to 112 due to selection and further fused to produce a total of 55 parameters. The selected and fused variables in this case included sex, country of birth, current depressive symptoms, ever smoked, family-related stress, other stress, participation in team sports and worry about weight (Table with results is in the main manuscript).

We also applied LTMLE to estimate the target parameters of the MSM. Based on the same outcome models which involved all covariate main terms and the interaction terms between sex and treatments, we implemented: 1) LTMLE using propensity scores on the full set of covariates; 2) LTMLE using propensity scores on the selected set of covariates by LOAL; 3) LTMLE using propensity scores after LOAL selection and fusion. In addition, we also applied LTMLE with machine learning (`superlearner R` package) for stratified treatment models, censoring models and outcome models in which we included

the algorithms: “SL.mean”, “SL.glm”, “SL.gam”, “SL.gam, screen.randomForest”, “SL.glm.interaction”, “SL.glm.interaction, screen.randomForest”, “SL.earth”, “SL.earth, screen.randomForest”. Estimated coefficients and standard errors are presented in the Table in the main manuscript. We used the `sandwich R` package to estimate the robust sandwich variance of IPTW and the variance of LTMLE was estimated based on influence function.

All methods consistently demonstrated that being female was associated with more severe depressive symptoms when compared to males, who served as the reference group. IPTW full, LTMLE full, and LTMLE SL had point-estimates that suggested that early alcohol initiation was linked to detrimental effects on depressive symptoms in males. All IPTW point-estimates suggested that earlier alcohol initiation was beneficial for females, while the LTMLE results indicated harmful or null effects for females. Furthermore, propensity scores derived from covariates selected by LOAL and then fused led to apparent reduced estimation variance in both IPTW and LTMLE analyses. In addition, to assess the extent of practical positivity violations, we examined the summary of the cumulative product probabilities of treatment and censoring under the full model and the model selected by LOAL, as used in the IPTW analyses. The results are presented in Table D1, showing that using full models and models selected by LOAL yield high medians near 0.90, with LOAL exhibiting slightly less extreme minimum and maximum values.

TABLE D1 NDIT analysis: Summary of the cumulative product probabilities of treatment and censoring using full models or with models selected by LOAL

Method	Min.	10%	25%	50%	75%	90%	Max.
Full	0.0008	0.1858	0.4595	0.9031	0.9720	0.9898	0.9991
LOAL	0.0012	0.1749	0.4409	0.9038	0.9590	0.9775	0.9893

TABLE D2 NDIT analysis: Grid of balance criteria values based on 20 values of λ^a and λ^c, used in the selection step for the treatment and censoring outcome-adaptive LASSOs. The first column represents 20 values of λ^a (treatment model) and the first row represents 20 values of λ^c (censoring model). In each cell, the left number represents the balance criterion value related to treatment and the right number represents the balance criterion value related to censoring for the corresponding λ^a and λ^c pair. The pair of numbers highlighted in red indicates minimum sum of the balance criteria.

	λ_n^a/λ_n^c	1<br>22026.466	2<br>10001.86	3<br>4541.682	4<br>2062.304	5<br>936.459	6<br>425.231	7<br>193.09	8<br>87.679	9<br>39.814	10<br>18.079
1	2980.958	(16.369, 46.532)	(16.369, 46.532)	(16.31, 46.578)	(16.377, 47.207)	(16.306, 48.167)	(16.292, 48.351)	(16.256, 48.497)	(16.218, 47.631)	(16.242, 47.788)	(16.181, 48.484)
2	1585.129	(14.432, 49.587)	(14.432, 49.587)	(14.389, 49.472)	(14.455, 48.651)	(14.334, 48.255)	(14.313, 48.281)	(14.272, 48.263)	(14.202, 47.601)	(14.196, 47.703)	(14.172, 47.805)
3	842.895	(13.629, 51.104)	(13.629, 51.104)	(13.593, 51.027)	(13.632, 50.253)	(13.504, 50.003)	(13.484, 50.035)	(13.45, 50.08)	(13.389, 49.672)	(13.398, 49.559)	(13.408, 49.453)
4	448.211	(13.517, 51.445)	(13.517, 51.445)	(13.482, 51.374)	(13.521, 50.695)	(13.394, 50.409)	(13.366, 50.433)	(13.331, 50.577)	(13.283, 50.157)	(13.294, 49.956)	(13.325, 49.929)
5	238.337	(12.925, 49.895)	(12.925, 49.895)	(12.886, 49.772)	(12.928, 49.003)	(12.882, 48.973)	(12.882, 49.058)	(12.85, 49.057)	(12.78, 48.514)	(12.734, 48.773)	(12.691, 48.791)
6	126.736	(13.011, 49.078)	(13.011, 49.078)	(12.974, 49.043)	(13.024, 49.144)	(12.987, 52.875)	(12.988, 53.166)	(12.977, 53.071)	(12.916, 52.259)	(12.863, 52.665)	(12.793, 53.189)
7	67.392	(13.233, 48.36)	(13.233, 48.36)	(13.196, 48.367)	(13.25, 47.889)	(13.138, 48.241)	(13.142, 48.33)	(13.128, 48.433)	(13.102, 47.836)	(13.078, 47.709)	(13.001, 47.548)
8	35.836	(13.769, 48.901)	(13.769, 48.901)	(13.729, 49.341)	(13.782, 48.709)	(13.8, 53.452)	(13.889, 53.535)	(13.923, 53.453)	(13.809, 52.908)	(13.757, 52.433)	(13.534, 53.065)
9	19.056	(12.23, 63.313)	(12.23, 63.313)	(12.182, 63.526)	(12.242, 65.978)	(12.221, 70.768)	(12.281, 70.792)	(12.3, 70.589)	(12.196, 69.671)	(12.147, 69.004)	(12, 69.429)
10	10.133	(14.396, 79.943)	(14.396, 79.943)	(14.352, 80.08)	(14.425, 81.565)	(14.339, 81.133)	(14.36, 81.155)	(14.372, 81.368)	(14.318, 82.405)	(14.272, 81.373)	(14.184, 82.627)
11	5.388	(13.033, 50.673)	(13.033, 50.673)	(12.976, 50.8)	(13.07, 51.984)	(12.953, 56.357)	(12.953, 56.32)	(12.931, 55.807)	(12.924, 54.88)	(12.896, 55.083)	(12.845, 55.337)
12	2.865	(11.737, 78.484)	(11.737, 78.484)	(11.681, 78.818)	(11.779, 78.971)	(11.66, 79.397)	(11.653, 79.338)	(11.63, 79.895)	(11.645, 79.97)	(11.624, 78.424)	(11.582, 79.129)
13	1.524	(10.21, 73.849)	(10.21, 73.849)	(10.159, 74.314)	(10.256, 74.081)	(10.101, 75.355)	(10.085, 75.27)	(10.057, 75.701)	(10.091, 76.048)	(10.08, 74.789)	(10.075, 75.687)
14	0.81	(10.125, 78.234)	(10.125, 78.234)	(10.069, 78.646)	(10.166, 77.191)	(10.069, 78.387)	(10.075, 78.158)	(10.062, 78.571)	(10.056, 79.251)	(10.035, 78.483)	(9.979, 78.849)
15	0.431	(9.926, 77.562)	(9.926, 77.562)	(9.872, 77.969)	(9.964, 76.484)	(9.85, 77.671)	(9.848, 77.439)	(9.835, 77.861)	(9.84, 78.573)	(9.815, 77.751)	(9.784, 78.117)
16	0.229	(10.198, 82.721)	(10.198, 82.721)	(10.137, 82.982)	(10.232, 82.354)	(10.147, 82.951)	(10.145, 82.762)	(10.127, 82.964)	(10.128, 83.23)	(10.105, 83.2)	(10.051, 83.456)
17	0.122	(9.681, 85.639)	(9.681, 85.639)	(9.626, 85.92)	(9.724, 85.554)	(9.629, 85.726)	(9.614, 85.573)	(9.587, 85.704)	(9.586, 85.678)	(9.571, 86.078)	(9.547, 86.678)
18	0.065	(10.333, 85.462)	(10.333, 85.462)	(10.278, 85.721)	(10.376, 85.195)	(10.271, 85.544)	(10.264, 85.396)	(10.238, 85.512)	(10.25, 85.467)	(10.238, 85.73)	(10.223, 86.233)
19	0.034	(10.376, 84.707)	(10.376, 84.707)	(10.32, 85.021)	(10.414, 84.404)	(10.314, 84.796)	(10.307, 84.648)	(10.285, 84.789)	(10.296, 84.762)	(10.288, 85.052)	(10.265, 85.627)
20	0.018	(9.842, 82.699)	(9.842, 82.699)	(9.786, 83.157)	(9.878, 82.589)	(9.775, 82.927)	(9.772, 82.786)	(9.763, 82.662)	(9.754, 82.957)	(9.747, 83.58)	(9.702, 84.764)

	λ_n^a/λ_n^c	11<br>8.209	12<br>3.728	13<br>1.693	14<br>0.769	15<br>0.349	16<br>0.158	17<br>0.072	18<br>0.033	19<br>0.015	20<br>0.007
1	2980.958	(16.241, 47.392)	(16.268, 47.381)	(16.304, 47.619)	(16.196, 48.132)	(16.202, 48.08)	(16.211, 48.124)	(16.206, 48.47)	(16.214, 48.083)	(16.251, 48.359)	(16.251, 48.362)
2	1585.129	(14.201, 47.442)	(14.22, 47.336)	(14.259, 47.534)	(14.135, 48.18)	(14.129, 47.388)	(14.139, 47.278)	(14.133, 47.424)	(14.139, 47.375)	(14.176, 47.953)	(14.176, 47.936)
3	842.895	(13.379, 49.057)	(13.4, 49.067)	(13.464, 49.183)	(13.317, 49.981)	(13.305, 49.313)	(13.313, 49.209)	(13.31, 49.466)	(13.312, 49.396)	(13.348, 49.893)	(13.349, 49.881)
4	448.211	(13.283, 49.489)	(13.306, 49.489)	(13.371, 49.691)	(13.218, 50.605)	(13.192, 49.802)	(13.2, 49.708)	(13.195, 49.853)	(13.189, 49.82)	(13.223, 50.437)	(13.224, 50.426)
5	238.337	(12.675, 48.203)	(12.691, 48.246)	(12.705, 48.394)	(12.634, 49.274)	(12.617, 48.925)	(12.625, 48.857)	(12.622, 48.992)	(12.618, 48.9)	(12.648, 49.573)	(12.649, 49.561)
6	126.736	(12.791, 51.678)	(12.804, 51.85)	(12.821, 51.639)	(12.757, 52.187)	(12.756, 53.047)	(12.765, 53.082)	(12.759, 53.512)	(12.753, 53.23)	(12.782, 53.336)	(12.782, 53.344)
7	67.392	(12.99, 47.300)	(13.009, 47.213)	(13.047, 47.37)	(12.941, 47.901)	(12.984, 47.415)	(12.991, 47.361)	(12.986, 47.613)	(12.994, 47.469)	(13.021, 47.791)	(13.02, 47.794)
8	35.836	(13.561, 52.193)	(13.559, 51.975)	(13.515, 51.59)	(13.477, 50.41)	(13.534, 51.194)	(13.541, 51.249)	(13.541, 51.16)	(13.558, 51.292)	(13.592, 50.791)	(13.588, 50.83)
9	19.056	(12.029, 68.203)	(12.035, 68.258)	(12.035, 67.988)	(11.94, 66.67)	(12.021, 67.544)	(12.028, 67.56)	(12.015, 68.187)	(12.028, 68.354)	(12.06, 68.077)	(12.058, 68.107)
10	10.133	(14.224, 81.347)	(14.235, 81.301)	(14.266, 81.324)	(14.184, 80.782)	(14.259, 80.037)	(14.267, 80.021)	(14.262, 80.026)	(14.272, 80.354)	(14.305, 80.168)	(14.302, 80.212)
11	5.388	(12.872, 54.095)	(12.895, 54.104)	(12.926, 53.702)	(12.87, 52.07)	(12.914, 53.444)	(12.919, 53.514)	(12.913, 53.945)	(12.92, 54.03)	(12.95, 53.677)	(12.949, 53.71)
12	2.865	(11.608, 79.496)	(11.64, 79.382)	(11.684, 79.465)	(11.583, 78.679)	(11.629, 78.595)	(11.634, 78.551)	(11.614, 78.531)	(11.618, 78.872)	(11.65, 78.364)	(11.647, 78.405)
13	1.524	(10.086, 75.889)	(10.114, 75.651)	(10.184, 75.592)	(10.051, 74.761)	(10.09, 74.363)	(10.095, 74.311)	(10.073, 74.413)	(10.075, 74.792)	(10.106, 74.1)	(10.103, 74.144)
14	0.81	(10.032, 79.517)	(10.051, 79.263)	(10.094, 79.325)	(9.983, 78.107)	(10.028, 78.435)	(10.033, 78.4)	(10.014, 78.353)	(10.019, 78.587)	(10.053, 78.176)	(10.049, 78.218)
15	0.431	(9.811, 78.784)	(9.837, 78.52)	(9.891, 78.6)	(9.79, 77.35)	(9.837, 77.661)	(9.843, 77.63)	(9.825, 77.613)	(9.818, 77.864)	(9.846, 77.454)	(9.843, 77.496)
16	0.229	(10.106, 83.874)	(10.115, 83.644)	(10.157, 83.668)	(10.042, 82.409)	(10.084, 82.672)	(10.09, 82.666)	(10.078, 82.637)	(10.073, 82.794)	(10.105, 82.672)	(10.102, 82.716)
17	0.122	(9.547, 86.89)	(9.548, 86.636)	(9.6, 86.675)	(9.535, 85.576)	(9.58, 85.272)	(9.589, 85.272)	(9.573, 85.225)	(9.549, 85.513)	(9.578, 85.992)	(9.576, 86.036)
18	0.065	(10.219, 86.494)	(10.223, 86.24)	(10.287, 86.264)	(10.184, 85.157)	(10.23, 84.971)	(10.239, 84.966)	(10.223, 84.91)	(10.207, 85.175)	(10.235, 85.479)	(10.233, 85.523)
19	0.034	(10.267, 85.835)	(10.268, 85.56)	(10.334, 85.58)	(10.242, 84.484)	(10.285, 84.148)	(10.294, 84.143)	(10.278, 84.112)	(10.262, 84.407)	(10.293, 84.797)	(10.291, 84.842)
20	0.018	(9.741, 84.284)	(9.741, 83.937)	(9.775, 83.957)	(9.733, 82.972)	(9.773, 82.141)	(9.781, 82.143)	(9.769, 82.121)	(9.756, 82.356)	(9.772, 83.187)	(9.77, 83.231)

REFERENCES

1. Jackson J. Diagnostics for Confounding of Time-varying and Other Joint Exposures. *Epidemiology.* 2016;27(6):859-869. Doi: 10.1097/EDE.0000000000000547.
2. Adenyo D, Guertin JR, Candas B, et al. Evaluation and comparison of covariate balance metrics in studies with time-dependent confounding. *Statistics in Medicine.* 2024;43(23):4437–4455.
3. Viallon V, Lambert-Lacroix S, Hoefling H, et al. On the robustness of the generalized fused lasso to prior specifications. *Statistics and Computing.* 2016;26:285-301. Doi: 10.1007/s11222-014-9497-6.
4. Viallon V, Lambert-Lacroix S, Höfling H, et al. Adaptive Generalized Fused- Lasso: Asymptotic Properties and Applications. 2013:hal-00813281.
5. Schnitzer M, Sango J, Ferreira Guerra S, et al. Data-adaptive longitudinal model selection in causal inference with collaborative targeted minimum loss-based estimation. *Biometrics.* 2020:145-157. Doi: 10.1111/biom.13135.
6. Gruber S, van der Laan MJ. An application of collaborative targeted maximum likelihood estimation in causal inference and genomics. *The International Journal of Biostatistics.* 2010;6(1).
7. Ju C, Gruber S, Lendle S, et al. Scalable collaborative targeted learning for high-dimensional data. *Statistical Methods in Medical Research.* 2019;28(2):532-554. Doi: 10.1177/0962280217729845.
8. Bickel PJ, Götze F, van Zwet WR. Resampling fewer than n observations: gains, losses, and remedies for losses. *Statistica Sinica.* 1997:1–31.
9. Politis DN, Romano JP. Large sample confidence regions based on subsamples under minimal assumptions. *The Annals of Statistics.* 1994:2031–2050.
10. Bickel PJ, Sakov A. On the choice of m in the m out of n bootstrap and confidence bounds for extrema. *Statistica Sinica.* 2008:967–985.
11. Bertail P, Politis DN, Romano JP. On subsampling estimators with unknown rate of convergence. *Journal of the American Statistical Association.* 1999;94(446):569–579.
12. Chakraborty B, Laber EB, Zhao Y. Inference for optimal dynamic treatment regimes using an adaptive m-out-of-n bootstrap scheme. *Biometrics.* 2013;69(3):714–723.
13. O'Loughlin J, Dugas EN, Brunet J, et al. Cohort profile: the nicotine dependence in teens (NDIT) study. *International Journal of Epidemiology.* 2015;44(5):1537–1546.
14. Cloninger CR. A systematic method for clinical description and classification of personality variants: A proposal. *Archives of General Psychiatry.* 1987;44(6):573–588.
15. Liu Y, Schnitzer ME, Herrera R, et al. The application of target trials with longitudinal targeted maximum likelihood estimation to assess the effect of alcohol consumption in adolescence on depressive symptoms in adulthood. *American Journal of Epidemiology.* 2024;193(6):835–845.
16. Rosenberg M. Rosenberg self-esteem scale (RSE). Acceptance and commitment therapy. Measures package, 61 (52), 18. *Wollongong, Australia: University of Wollongong.* 1965.
17. Racicot S, McGrath JJ, Karp I, et al. Predictors of nicotine dependence symptoms among never-smoking adolescents: a longitudinal analysis from the Nicotine Dependence in Teens Study. *Drug and Alcohol Dependence.* 2013;130(1-3):38–44.
18. Eysenck SB, Eysenck HJ. Impulsiveness and venturesomeness: Their position in a dimensional system of personality description. *Psychological reports.* 1978;43(3_suppl):1247–1255.
19. Wills TA, Windle M, Cleary SD. Temperament and novelty seeking in adolescent substance use: convergence of dimensions of temperament with constructs from Cloninger's theory. *Journal of Personality and Social Psychology.* 1998;74(2):387.
20. Otter C, Huber J, Bonner A. Cloninger's Tridimensional Personality Questionnaire: reliability in an English sample. *Personality and Individual Differences.* 1995;18(4):471–480.
21. Chaiton M, Contreras G, Brunet J, et al. Heterogeneity of depressive symptom trajectories through adolescence: Predicting outcomes in young adulthood. *Journal of the Canadian Academy of Child and Adolescent Psychiatry.* 2013;22(2):96–105.
22. Escobedo LG, Kirch DG, Anda RF. Depression and smoking initiation among US Latinos. *Addiction.* 1996;91(1):113–119.

23. Bech P, Stage K, Nair N, et al. The Major Depression Rating Scale (MDS). Inter-rater reliability and validity across different settings in randomized moclobemide trials. *Journal of Affective Disorders.* 1997;42(1):39–48.

24. Bech P, Timmerby N, Martiny K, et al. Psychometric evaluation of the Major Depression Inventory (MDI) as depression severity scale using the LEAD (Longitudinal Expert Assessment of All Data) as index of validity. *BMC Psychiatry.* 2015;15(1):1–7.

25. Van Buuren S, Groothuis-Oudshoorn K. mice: Multivariate imputation by chained equations in R. *Journal of Statistical Software.* 2011;45:1–67.

26. Shortreed S, Ertefaie A. Outcome-adaptive lasso: Variable selection for causal inference. *Biometrics.* 2017;73(4):1111-1122. Doi: 10.1111/biom.12679.
